# Financial accounting for deferred taxes: a systematic review of empirical evidence

**DOI:** 10.1007/s11301-021-00233-w

**Published:** 2021-09-27

**Authors:** Anna Görlitz, Michael Dobler

**Affiliations:** Chair of Accounting, Auditing and Taxation, Technische Universität Dresden, Dresden, Germany

**Keywords:** Deferred taxes, Earnings management, Income taxes, Value relevance, International Financial Reporting Standards, US Generally Accepted Accounting Principles, H25, M41, M48

## Abstract

Deferred taxes—resulting from differences between financial and tax accounts—have been a long-standing, contentious issue in financial accounting regulation, practice, and research. Debates on concepts and standards have been accompanied by doubts around whether and the extent to which deferred taxes provide relevant information for financial statement users and are employed by firms to manage their earnings. This paper systematically reviews the body of empirical evidence that has emerged over the last three decades on deferred taxes in the fields of value relevance and earnings management. A bibliographic analysis and a narrative synthesis are presented within a thematic categorization framework. Key results indicate that existing research focuses on the US setting. There is substantial evidence for the value relevance of various deferred tax items but limited evidence that firms use deferred taxes to manage their earnings. The findings suggest implications for both future research and the regulatory debate.

## Introduction

Despite some interrelations, tax accounting and financial accounting differ in their objectives, regulations, underlying incentives, as well as their amounts of assets, liabilities, and profits. Deferred taxes are a result of differences between tax accounts and financial accounts, and they have been subject to controversy and debates on concepts and standards around the world (Brouwer and Naarding 2018; Morton 2019; IASB 2018). As of today, financial accounting regulators have reached considerable consensus on fundamental concepts, such as considering deferred taxes as a result of temporary differences between the tax basis and the accounting basis of assets and liabilities. However, as standard-setters strive to enact standards that enhance the informativeness of financial statements, they struggle with the complexity of financial accounting for deferred taxes, as reflected in ongoing debates at national and international levels (FASB 2015; EFRAG 2019; IASB 2019, 2020). Likewise, financial accounting enforcement bodies (such as the US Securities and Exchange Commission and the German Financial Reporting Enforcement Panel) consider deferred taxes as a key source of potential errors in financial statements (DPR 2015; EY 2018).

The very nature of financial accounting for deferred taxes is forward-looking. As deferred taxes indicate probable future income tax deductions or burdens and assist financial statement users in assessing the financial position of a firm or group, financial accounting for deferred taxes is inevitably subject to estimation uncertainty and managerial judgment (Brouwer and Naarding 2018). This tension fosters the debate in financial accounting whether deferred taxes are value relevant or whether they are an instrument or indicator of earnings management. The value relevance of financial accounting items are defined as “the ability of financial statement information to capture and summarise information that determines the firm’s value” (Beisland 2009). Managerial judgment plays a crucial role in the informativeness of deferred taxes. Managerial judgment can be used either to communicate private information (relevant to financial statement users) or for window dressing purposes (with a potentially diluting effect on value relevance). The latter relates to earnings management which can be defined as the exploitation of managerial judgment to reach specific earnings targets (Healy and Wahlen 1999; Barth et al. 2001a).

Financial accounting regulators are aware of this tension. For example, the International Accounting Standards Board (IASB) states: “[U]sers are sceptical about deferred tax accounting because they do not understand what information is provided … and they suspect that deferred tax accounting is utilised to manage earnings” (IASB 2016a). This assessment emphasizes that financial accounting for deferred taxes may lead to little relevant information being provided to users of financial statements while offering substantial room for earnings management by preparers of financial statements (PWC 2016; FASB 2019).

The body of research on financial accounting for deferred taxes reflects this tension. While there is substantial evidence suggesting that deferred taxes are value relevant (e.g., Hanlon et al. 2014; Bauman and Shaw 2016; Johnston and Kutcher 2016), the findings on which items of deferred taxes are value relevant are mixed and differ across research methods and institutional settings. In turn, for example, there is evidence that large amounts of deferred taxes signal low earnings quality, earnings management, and are related to higher audit cost, as they are considered to be a ‘red flag’ (e.g., Hanlon 2005; Crabtree and Maher 2009; Blaylock et al. 2012).

Against this backdrop, our paper systematically reviews empirical studies in financial accounting that focus on value relevance and/or earnings management in the context of deferred taxes to assess two related questions:

- To what extent does empirical research suggest that accounting for deferred taxes is value relevant as a key facet of informativeness for financial statement users?
- To what extent does empirical research suggest that accounting for deferred taxes relates to earnings management?

This review focuses on research published in refereed academic journals with a high-quality ranking according to SCImago journal ranks (SCImago 2019) and covers 76 research articles published over three decades. Based on Tranfield et al. (2003), Fisch and Block (2018), Block and Fisch (2020), and Clark et al. (2020), we provide both a bibliographic analysis and a narrative synthesis. Our paper distinguishes itself from existing reviews in both (1) the more general field of accounting for income taxes, and (2) the field of financial accounting for deferred taxes in particular. Reviews of the first strand cover deferred taxes as one specific aspect among many. For example, Graham et al. (2012) suggest a general framework for income tax accounting literature, and Hanlon and Heitzman (2010) rather focus on the role of taxes in real business decisions. Our review also goes well beyond existing reviews of the second strand. Brouwer and Naarding (2018) provide a literature review on issues of relevance of deferred tax accounting that is rather focused on analytical and theoretical work, and Breitkreuz (2012) provides a semi-structured review of empirical work in the German language.

Our review, thus, contributes in several ways. First, the major reviews to date only cover empirical studies published up to 2010. In contrast, almost half of the empirical studies discussed in our paper have been published since 2010. Second, most studies published up to 2010 relate to US samples. In contrast, our review includes novel empirical evidence beyond the US setting. In addition, our review is the first in the field to provide a bibliographic analysis. As a result, the main contribution of our review is a systematic survey of the body of empirical evidence that has been built up to 2019. In the course of our review, we identify research gaps that offer avenues for future research, and we indicate some implications for the regulatory debate on financial accounting for deferred taxes.

The remainder of this paper is structured as follows. Section 2 summarizes the regulatory background on financial accounting for deferred taxes. Section 3 describes the relation between deferred taxes, value relevance, and earnings management. Sections 4 and 5 present the research methodology and categorization framework. Section 6 describes and discusses the results of the bibliographic analysis. Section 7 provides the narrative syntheses on value relevance studies and earnings management studies. Section 8 presents implications for future research, followed by a concluding Sect. 9.

## Overview of the rules governing accounting for deferred taxes

Conceptually, deferred taxes are a result of differences in tax profit and accounting profit under the income statement approach, or of differences in the tax basis and the accounting basis of assets and liabilities under the balance sheet approach (Dichev 2008; Brouwer and Naarding 2018). Contemporary regulations on accounting for deferred taxes tend to follow the balance sheet approach to provide a true and fair view of assets and liabilities in financial statements. These regulations consider temporary book-tax differences regardless of the estimated period until the differences reverse (temporary concept), and they value the differences at the future tax rate at the time of reversal (liability method). Thus, deferred tax assets as probable future tax deductions contribute positively to firm value, while deferred tax liabilities contribute negatively to firm value (Legoria and Sellers 2005; Graham et al. 2012).

The current regulations on accounting for deferred taxes under US GAAP, IFRS, and many national accounting systems have their origins in the late 1980s in the US (Schultz and Johnson 1998; Harumova 2017). Deferred tax accounting played a minor role in accounting practice before the balance sheet approach was introduced in 1992 by SFAS No. 109 (ASC 740, according to FASB’s codification system) (Legoria and Sellers 2005). The prior US GAAP standard APB No. 11 followed the income statement approach and consisted of restrictive rules on the recognition and measurement of deferred tax assets; therefore, the share of deferred tax expenses in the income statement was small. SFAS No. 96 superseded APB No. 11 and introduced the balance sheet approach, but its adoption was never formally required, due to its overly complex nature. Thus, the adoption of SFAS No. 109 in 1992 was well accepted by US practice (Ayers 1998; Schultz and Johnson 1998); it led to increased recognition of deferred tax assets in financial statements under US GAAP (Poterba et al. 2011) and stimulated regulatory attempts internationally.

In 1994, the predecessor body of the IASB published the exposure draft of a fully-revised IAS 12. This standard was formally adopted in 1996. Since then, the regulations governing deferred tax accounting under US GAAP (SFAS No. 109, ASC 740) and IFRS (IAS 12) have been rather similar. The fundamental accounting principles—such as the balance sheet approach, the liability method, and the temporary concept—were further adopted in other national accounting standards, including Australian GAAP (effective date 2005), German GAAP (effective date 2009), and UK GAAP (effective date 2015). Table 1 compares the standards on accounting for deferred taxes to which the existing empirical research reviewed in this paper most frequently refers. The current rules under UK GAAP and German GAAP on accounting for deferred taxes are similar to those under IFRS and Australian GAAP.

**Table 1:** Comparison of selected accounting standards on deferred taxes

Category	US GAAP	IFRS	Australian GAAP
Standard	ASC 740	IAS 12	AASB 112
Year of adoption	1992	1996	2005
Delimitation approach (temporary vs**.** timing concept)	Temporary concept, ASC 740-10-25-2b	Temporary concept, IAS 12.15 and IAS 12.24	Temporary concept, AASB 112.15 and AASB 112.24
Allocation procedure (balance sheet vs. income statement approach)	Comprehensive allocation, ASC 740-10-30-5 and ASC 740-10-25	Comprehensive allocation, IAS 12.15 and IAS 12.24	Comprehensive allocation, AASB 112.15 and AASB 112.24
Valuation method (liability vs. deferred method)	Liability method	Liability method	Liability method
Recognition approach	Impairment approach	Affirmative judgement approach	Affirmative judgement approach
Recognition of DTA without VA	Yes, ASC 740-10-50-2a	No	No
Realisation probability	50%, ASC 740-10-25	50%, IFRS 5 BC81	50%, AASB 12.24
Recognition of the VA	Yes, ASC 740-10-50-2c	No	No
Recognition of DTL	Yes, ASC 740-10-50-2a	Yes, IAS 12.81 (g) (i)	Yes, AASB 112.81 (g) (i)

*DTA* deferred tax assets, *DTL* deferred tax liabilities, *VA* valuation allowance

Differences between US GAAP and other standards relate to the valuation method and the recognition approach. For example, the applicable tax rate for valuing deferred taxes differs: US GAAP (ASC740-10-30-8) require using enacted tax rates, whereas IFRS (IAS 12.47) and Australian GAAP (AASB 112.47) allow referring to announced tax rates that are not enacted at the time when the financial statements are prepared. Moreover, the recognition approach is different under US GAAP, which follows the impairment approach, while other standards follow the affirmative judgment approach. The affirmative judgment approach allows recognizing deferred assets to the extent that it is probable that taxable profit will be available in the future (IAS 12.24); as a result, no valuation allowance is needed. In contrast, the impairment approach under US GAAP requires first the recognition of deferred tax assets for all temporary deductible differences. In a second step, a valuation allowance to reduce deferred tax assets is recognized based on the available evidence that some or all of the deferred tax assets will not be realized in the future (Harumova 2017).

In the past, the principles of accounting for deferred taxes were led by US GAAP. For example, the IASB published an exposure draft in 2009 (ED/2009/2) that proposed adopting the US impairment approach (ED/2009/2.5 and 2.23) to further harmonize accounting systems internationally. While overall critical feedback on this exposure draft stopped the IASB project, feedback largely favored the impairment approach instead of the affirmative approach under IAS 12. Still, an introduction of the impairment approach by the IASB is under discussion (EFRAG 2013). In the US, the recently updated accounting standard (ASU 2015-17) classifies all deferred taxes as noncurrent items, similar to IFRS and Australian GAAP. This is a rare example where US GAAP adopts rules in IFRS and other national GAAP. While harmonization of GAAP that governs accounting for deferred taxes is still on the way, recent GAAP in jurisdictions covered by the empirical studies reviewed in this paper shares common concepts despite some particularities in US GAAP.

## Value relevance and earnings management in context

It is a generally accepted objective of financial statements to provide relevant information that is useful to take economic decisions (Barth et al. 2001b; Brouwer and Naarding 2018). According to the US FASB, financial statements shall “provide information that is useful to present and potential investors and creditors and other users in making rational investment, credit, and similar decisions” (SFAC No. 1 para 34). The information provided should be useful in assessing the amounts, timing, and probability of future cash flows (SFAC No. 1 para 37). Such regulatory requirements translate into the value relevance of financial statement information. Value relevance is a widely employed, well-faceted concept in empirical accounting research and can be defined as “the ability of financial statement information to capture and summarise information that determines the firm’s value” (Beisland 2009). Value relevance is the first concept that we address in our review.

Despite specific financial accounting standards in place, financial accounting for deferred taxes offers substantial room for discretion. This discretion particularly relates to the forward-looking nature of financial accounting for deferred taxes that inevitably requires managerial judgment (Graham et al. 2012; Brouwer and Naarding 2018). Classical agency theory suggests that financial accounting is a means to reduce information asymmetry between corporate management (as preparers of financial statements) and corporate stakeholders (as users of financial statements) (Fama 1980; Coase 1990; Lambert 2006). Financial accounting literature indicates that managerial judgment is a double-edged sword the use of which depends on managerial incentives (Feltham and Pae 2000; Sankar and Subrahmanyam 2001). That is, management can exercise judgment for information communication or opportunistic purposes.

In the first case, managerial judgment is employed to communicate private information to financial statement users. Private information that is reflected in deferred tax items and considered reliable is likely to enhance the value relevance of deferred taxes. In the second case, managerial judgment is employed for opportunistic purposes, such as window dressing through earnings management. Earnings management can be defined as the use of managerial discretion “to alter financial reports to either mislead some stakeholders about the underlying economic performance of the company or to influence contractual outcomes that depend on reported accounting numbers” (Healy and Wahlen 1999). Earnings management is the second concept that we address in our review.^1^

Earnings management and value relevance are related. First, financial accounting research suggests that the higher the value relevance of an accounting variable, the higher is the earnings quality, and the lower is the probability that earnings are managed towards a certain target (Ewert and Wagenhofer 2013). Second, there seems to be consensus in financial accounting literature that opportunistic use of managerial judgment through earnings management is likely to dilute the value relevance of financial accounting information (Guay et al. 1996; Sankar and Subrahmanyam 2001; Rego and Wilson 2012). As emphasized before the tension between conveying relevant information and opportunistic earnings management purposes fosters the debate on financial accounting for deferred taxes (IASB 2016a; FASB 2019). Therefore, it is important to understand the information inherent in deferred taxes as reflected in both, value relevance studies and earnings management studies.

## Research methodology

The aim of this review is the systematic analysis and synthesis of the deferred tax literature, with a focus on value relevance and earnings management studies. The definition and the delimitation of the research area for compiling and synthesizing a comprehensive sample of studies build on the methodology of Tranfield et al. (2003) for systematic literature reviews and the journal-specific methodology guidelines of Fisch and Block (2018) and Clark et al. (2020). Transparency, reproducibility, and systematics are general quality criteria for a systematic review (Denyer and Tranfield 2009). The applied methodology for systematic literature review consists of three main stages: planning, conducting, and reporting. The planning stage aims to define the research area and the need for research, based on initial unsystematic literature research. The result of this stage is the a priori research strategy, which summarizes various qualitative and content-related inclusion and exclusion criteria. Based on the a priori search strategy, search strings are defined and applied in various databases to identify topic-specific studies. Additional search methods complement the search results. Synthesizing the relevant studies is the last step of the conducting stage. The reporting stage reports and critically evaluate the results to contribute meaningful findings to the research area (Tranfield et al. 2003).

Three independent search methods ensure identifying a representative population of articles: the Boolean search through electronic databases, the targeted journal search through leading journals, and the backward search (Hart 2001; Tranfield et al. 2003). For an article to be included in the review, it must meet two requirements. First, it must be an empirical study that includes regression analyses. Second, it must be published in a high-quality academic journal. To guarantee the quality of the journals and thereby the quality of the articles included in the review, we use strict cut-off criteria based on the SCImago journal ranking (SJR). We exclude all articles published in journals without SJR quality category (Q1; Q2; Q3; Q4) or in the lowest SJR decile of our sample (SJR < 38). All articles in the lowest quartile (SJR < 306) and all articles from journals from the lowest quality category (Q4) were screened and eventually excluded. These two criteria omit purely descriptive empirical studies and ensure an acceptable standard of scientific quality in the studies included in this review.

**Fig. 1:**
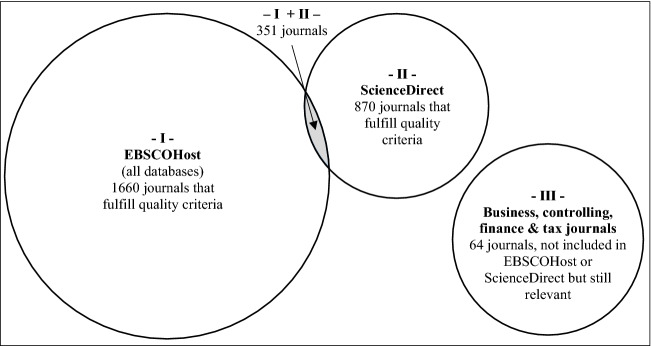
Venn diagram of journals analyzed

Figure 1 presents the number of journals included in the search process. The database EBSCOHost provides 2011 journals (I), and the database ScienceDirect provides 1221 journals (II) with 351 journals included in both databases. To further increase the number of journals searched, we identified 64 relevant high-quality journals that deal with business, controlling, finance, and tax topics and are not included in the databases EBSCOHost and ScienceDirect (III). Further search steps, such as the backward search, increases only slightly the number of journals searched through.

In addition to a large number of journals searched through, general keywords in English increase the possibility of identifying relevant studies. These keywords are “deferred tax*,” “book-tax difference*”, and “valuation allowance”. The Boolean search applied the keywords to the areas: title, abstract, and subject terms of the respective articles. The targeted journal searches and the backward search analyzed the title of potentially relevant articles. In all three cases, the selection focuses on academic publications in English. No other formal search restrictions were included.

**Fig. 2:**
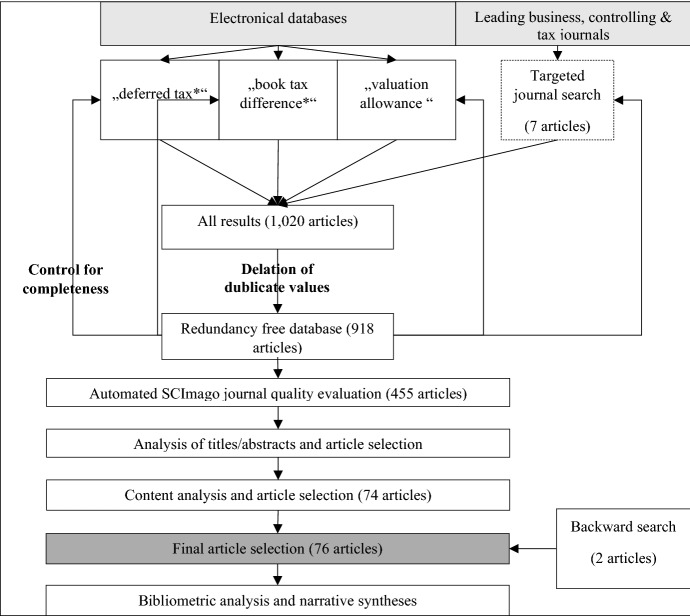
Process of article selection

The result of the search methods is a redundancy-free, topic-specific database with information about the author, the year of publication, the journal, its ISSN, and the abstract of each identified article. After the journal quality evaluation, we retain 455 of 918 articles that meet the quality criteria. The decision to include or exclude an article in the final sample depended on the a priori defined criteria. Consequently, 204 articles are excluded because they are non-empirical or do not include regression analyses. Other reasons for exclusion are that the studies do not focus on earnings management or value relevance related to deferred taxes (139 articles), and that the empirical study design only included total book-tax differences with no separate deferred tax variable (38 articles). The result of the search process is a list of 74 relevant studies. In the last step, the bibliographies of these 74 studies are analyzed using the backward search method, yielding 2 additional studies. The final sample contains 76 articles published between 1972 and 2019 in high-quality academic journals. Figure 2 displays the whole search process.

## Article categorization framework

We focus on two strands of empirical research on accounting for deferred tax—i.e., research related to value relevance and research related to earnings management—and use a content-driven categorization framework to structure the body of research. Other minor strands of research discuss regulatory implications of deferred tax accounting (e.g., Haupt and Ismer 2013; Eiler and Kutcher 2016), tax aggressiveness and the resulting book-tax differences (e.g., Lennox et al. 2013; Neifar and Utz 2019), and the impact of mandatory audits on deferred taxes (e.g., Kraft and Lopatta 2016; Laurion et al. 2017). The latter strands of research are not included in this review.

Value relevance studies investigate whether financial reporting and deferred tax accounting especially, provide forward-looking and decision-useful information for stakeholders and shareholders (Barth et al. 2001a). A great variety of empirical studies discuss the value relevance of deferred taxes, with most of them estimating variations of the following equation:

$$ {\text{Y}} = \beta_{0} + \beta_{1} DT + \beta_{2} \ldots \beta_{n} CV + \varepsilon $$

where Y represents an economic performance variable regressed on a deferred tax variable (DT) and a set of control variables (CV), including an error term ε.

Value relevance studies employ different types of performance variables. Based on Beaver (2002), this review clusters the value relevance studies according to the performance variable that is employed, with the categories of ‘forecast relevance studies’ and ‘valuation relevance studies’.

Forecast relevance studies assess the relation between deferred taxes and cash flow-relevant accounting variables, including cash flows, earnings, and income tax payments (Beaver 2002). International deferred tax accounting follows the balance sheet approach in combination with the liability view by classifying deferred tax liabilities (deferred tax assets) as liabilities (assets). These items contribute positively (negatively) to the firm value and, therefore, to the future cash flow (Harumova 2017). Due to the uncertain realization of deferred taxes, proponents of the liability view argue that related cash flows are highly ambiguous, with a present value close to zero. As most deferred taxes arise from operating and periodically recurring activities, newly-emerged temporary differences in the same fiscal year replace single reversing temporary differences directly. As a result, the replacement of reversed deferred taxes by new deferred taxes delays the final reversal of deferred taxes indefinitely, thereby throwing the value relevance of deferred taxes into question (IASB 2016b).

**Fig. 3:**
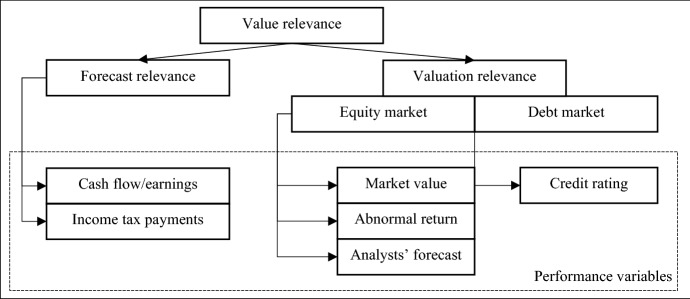
Categorization of value relevance studies

Valuation relevance studies assess how the information provided by deferred taxes is implemented in the decision-making processes of various financial statement users (Barth et al. 2001b; Beaver 2002). If deferred taxes meet the requirements of the liability view, the fundamental requirement for consideration by the capital markets and its value relevance is met (Barth et al. 2001b). Capital markets considered include the equity market and the debt market. Figure 3 shows the categorization of value relevance studies according to the performance variables employed.

Given the forward-looking nature of deferred taxes, deferred tax accounting inevitably offers opportunities for earnings management. Earnings management research in the field analyzes whether deferred taxes are an instrument for or an indicator of earnings management. Studies use various types of earnings management measures based on accruals, thresholds, or mis-/re-statements (Dechow and Skinner 2000; Dichev et al. 2013).

**Fig. 4:**
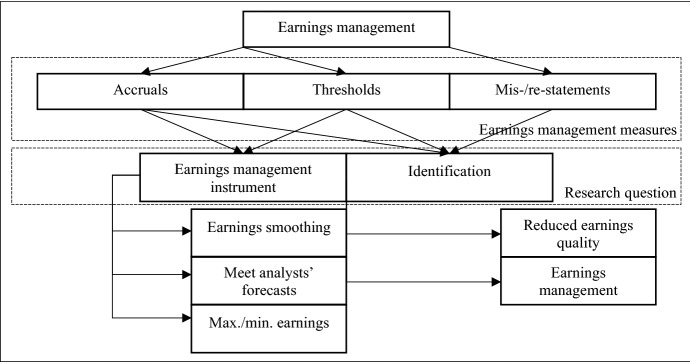
Categorization of earnings management studies

Mis-/re-statements provide information about the extent and the circumstances of opportunistic accounting by a certain firm. Research based on mis-/re-statements investigates whether deferred taxes are an indicator of earnings management (Frank and Rego 2006; Ettredge et al. 2008). The use of thresholds can reveal incentives to manage or smooth earnings upwards, downwards, or toward analysts’ forecasts. Accruals are a mathematical estimator for earnings management; they are calculated as the difference between net profit and cash flow from operating activities. The remaining non-cash income and expenses (accruals) can be divided (based on approximation procedures) into normal and discretionary accruals, where discretionary accruals are defined as results of earnings management (Jones 1991; Healy and Wahlen 1999). Figure 4 shows the categorization of earnings management studies according to the research questions and the earnings management measures that are employed.

## Bibliographic analysis

The bibliographic analysis of the 76 selected articles that aims to identify the structure and development of the research field is based on Zupic and Čater (2015) and Block and Fisch (2020). The quantitative approach can identify correlations, developments, and potential biases in a certain field of research (Zupic and Čater 2015). This section contains a topic analysis, a publication trend analysis, an affiliation analysis, and a citation analysis.

### Topic analysis

**Fig. 5:**
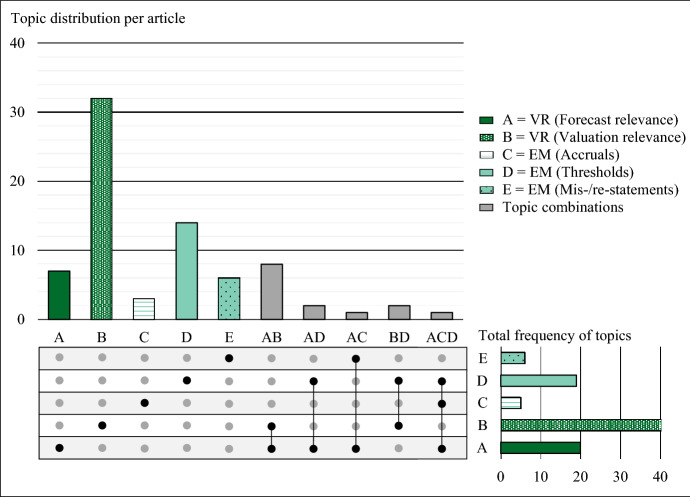
Topic distribution

Thematically, the major streams of deferred tax literature consist of value relevance studies—with subcategories focusing on forecast relevance and valuation relevance—and earnings management studies—with subcategories related to accruals, thresholds, and mis-/re-statements. As presented in Fig. 5, 62 of the 76 studies exclusively analyze one of these subcategories, 13 studies analyze two subcategories, and one study analyzes three subcategories. 6 out of 76 studies study both value relevance and earnings management. Most studies (53) analyze the value relevance of deferred taxes (A; B). The second main research topic is earnings management as operationalized by thresholds (D).

### Publication trend analysis

**Fig. 6:**
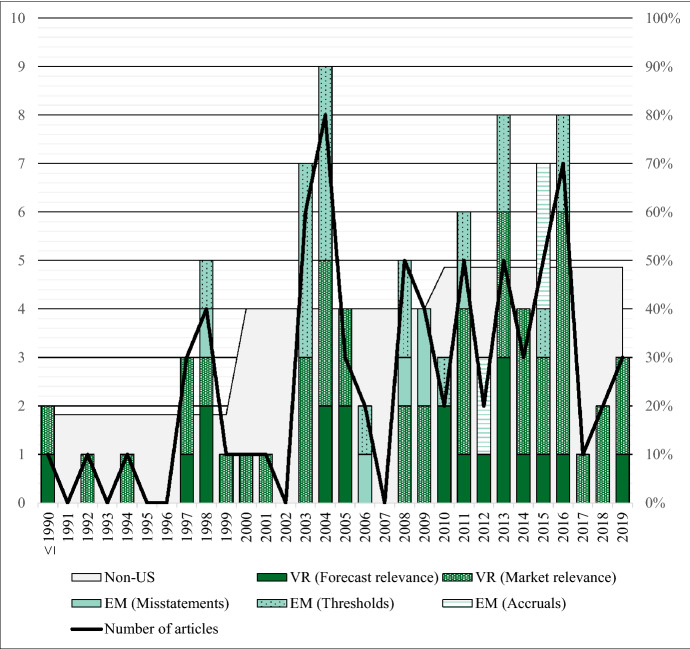
Year-wise publication trend analysis

The publication trend analysis evaluates the number of published articles per year, along with article specifications (e.g., the origin of the author) and sample specifications (e.g., the number of observed years and firm-years by the individual studies). Thus, single articles have multiple topic categorizations and appear twice or three times in the chart. Therefore, the number of articles (88) presented in the chart is larger than the absolute number of analyzed articles (76), due to these multiple categorizations. Figure 6 shows how the number of publications has developed over the three decades under analysis.

The total number of published articles is volatile and indicates an upward trend until 2016 and a downward trend for the years thereafter. The years 2004 and 2016 show the highest absolute numbers of publications. As the number of published articles per year is highly volatile, the below-average publication figures for 2017–2018 do not necessarily indicate a permanent decline in research interest; indeed, the absolute number of publications for 2019 is already increasing. The number of earnings management studies follows a similar course. All earnings management studies are published between 1998 and 2016. The number of earnings management studies rose sharply in the years 2003–2016: they account for more than 40% of the studies over the entire observation period. As the thematic categorization is based on the applied regression model, any value relevance study that only discusses the potential relation between deferred taxes and earnings management and that does not apply a separate regression model is not counted separately as an earnings management study. In this categorization, empirical research on deferred taxes that investigates earnings management is about as frequent as value relevance studies. Unlike the other trends, the number of value relevance studies increases rather steadily over the observed period. Furthermore, the number of non-US authors increases steadily over the observed period up to 48%.

The discussion, announcement, and implementation of SFAS No. 109 (ASC 740) triggered the initial research interest of US scholars at the beginning of the 1990s. In international accounting practice, deferred taxes played a minor role at this time; thus, US scholars started the scientific debate on whether the new standard leads to value relevant information. The debate resulted in the publication of value relevance studies during the first observation period (1990–2000); 90% of these early studies investigate US firms, and 82% of these studies have US authors. In the second and third observation decades (2001–2010; 2011–2019) the portion of non-US scholars steadily rose to 48%. This trend is consistent with the increasing importance of deferred taxes in international accounting practice, accompanied by regulatory changes (see Sect. 2).

In the second (2001–2010) and third decades (2011–2019), further research was triggered by high-profile accounting scandals like Enron, WorldCom, or Xerox, as well as the economic crisis of 2008 and various regulatory changes. Furthermore, researchers were motivated by the ongoing debate among preparers, regulators, standard-setters, and financial statement users as to whether any benefit in deferred tax accounting could justify the relatively high accounting costs (Beechy 2007; Colley et al. 2009; Bauman and Shaw 2016; Brouwer and Naarding 2018). These triggers motivated both earnings management studies and value relevance studies.

The average observation period and the average number of firm-year observations increases over the last three decades. Due to improved data accessibility and the harmonization of international accounting standards, larger samples, as well as cross-country studies are now possible.

### Affiliation analysis

We perform an affiliation analysis to identify top contributing journals and countries. There are 7 top contributing journals with at least three published articles. Table 2 shows the top contributing journals (in descending order) based on the number of published articles. The first authors of the analyzed studies originate from 13 countries. Table 3 provides a list of the origins of the first authors based on the number of published articles.

**Table 2:** Top contributing journals

Journal	SCImago quality	SJR	H Index	Number of articles	Origin
Accounting review	Q1	5446	143	13	US
Journal of the American Taxation Association	Q1	1468	20	12	US
Journal of Business Finance and Accounting	Q1	874	72	9	UK
Contemporary Accounting Research	Q1	2207	90	4	US
Accounting and Finance	Q2	43	44	3	US
Corporate Ownership and Control	Q4	148	18	3	Ukraine
Journal of Accounting Research (Wiley-Blackwell)	Q1	6996	132	3	UK

SCImago Quality: “The set of journals have been ranked according to their SJR and divided into four equal groups, four quartiles. Q1 (green) comprises the quarter of the journals with the highest values, Q2 (yellow) the second highest values, Q3 (orange) the third highest values and Q4 (red) the lowest values.” (Scimago 2019)

**Table 3:** Top contributing countries

Country	Number of articles	% of Articles
US	45	59
Australia	6	8
UK	5	7
Hong Kong	4	5
Canada	3	4
Brazil	2	3
Germany	2	3
Greece	2	3
New Zealand	2	3
Taiwan	2	3
Belgium	1	1
Japan	1	1
South Africa	1	1

The articles are highly concentrated in a small number of journals, as 62% of the articles are published in only 7 different journals. The SCImago journal quality rank rates 5 of these top contributing journals as Q1, one as Q2, and only one as Q4 indicating that the topic is frequently discussed in high-quality accounting and taxation journals (SCImago 2019). The countries of origin of the top contributing journals reflect the leading role of US journals, as three journals that contribute the most are US-based. In a similar vein, most first authors are from the US, further indicating the leading role of US research in the field. The overall concentration on common law countries—such as the US, Australia, and the UK—and financial statements based on common law-based standards—such as US GAAP and IFRS—seems to coincide with large amounts of deferred taxes in these settings (Atwood et al. 2010).

### Citation analysis

**Fig. 7:**
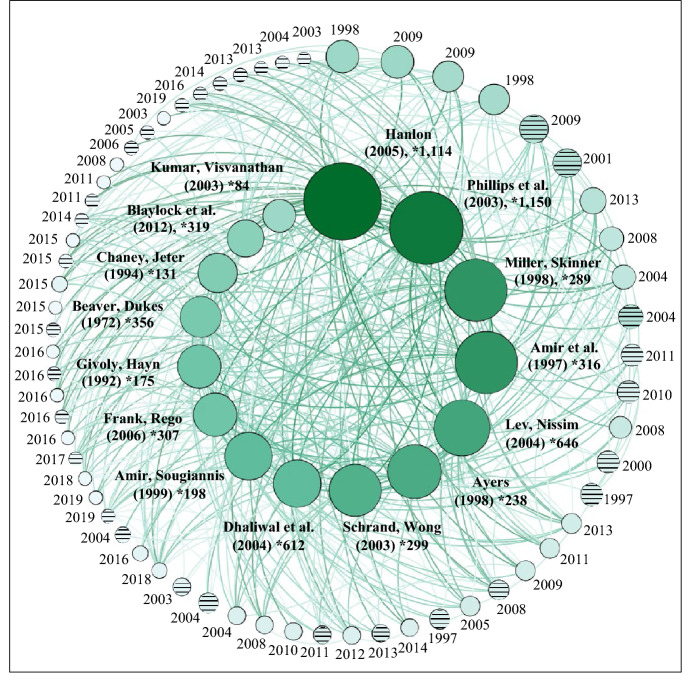
Citation analysis

A citation analysis identifies the impact and popularity of an article in the scientific community by counting the number of times other articles cite it (Zupic and Čater 2015). Our citation analysis covers all 76 articles identified. We find no substantial differences between the citation patterns of value relevance studies and those of earnings management studies. Figure 7 presents the results.

In Fig. 7, each bubble presents an article, falling in one of two circles depending on the citation frequency. Patterned bubbles represent non-US publications, while monochrome bubbles represent US publications. A line between two articles illustrates a citation. The inner circle displays the 15 most influential articles, which are cited between 9 and 28 times by the analyzed studies. The outer circle shows articles that are cited up to 8 times. Every article cites at least one article in the sample, indicating a coherent and strongly connected field of research. In the inner circle, 7 of the 15 studies are published in the first decade of investigation (≤ 1990–2000), and another 7 articles are published in the second decade of investigation (2001–2010). Only one article is published in the latest decade (2011–2019). US scholars have written all of the 15 most-cited articles, indicating the strong US influence in the research field.^2^

### Summary of the results from the bibliographic analysis

The bibliographic analysis reveals that the number of articles concerning deferred tax accounting has increased over the last three decades. The research focuses predominantly on value relevance questions; earnings management issues are underrepresented, especially in the first decade (1990–1999). In the second and third decades (2000–2019), the number of earnings management studies increases.

The proportion of non-US authors increases as well, as deferred tax accounting has become an essential topic in international accounting practice. However, the results of the citation analysis determine a citation bias towards older US publications. Two factors can mostly explain this bias. First, the leading role of US GAAP in developing and introducing accounting principles regarding deferred taxes leads to an early interest in US-focused deferred tax research. National standards (e.g., German GAAP, Australian GAAP, or UK GAAP) and international standards (IFRS) follow US deferred tax accounting principles (see Sect. 2). Second, a citation analysis generally involves a bias toward earlier publications (Zupic and Čater 2015). Frequently-cited early studies (e.g., Amir et al. 1997; Ayers 1998; Miller and Skinner 1998; Amir and Sougiannis 1999) analyze the potential one-time effects of the introduction of SFAS 109, which may have limited future significance. The use of similar samples by these and other early US studies overstates the described effect.

## Narrative synthesis

Our narrative synthesis of the 76 selected articles aims to synthesize and compare the evidence of the individual studies. According to Clark et al. (2020), the main outcome of a literature review is a well-structured summary that provides a foundation for future studies. Therefore, we base our synthesis on the categorization framework presented in Sect. 5.

### Narrative synthesis of the value relevance studies

According to the categorization framework, value relevance studies can be divided into forecast relevance studies and valuation relevance studies.

Forecast relevance studies analyze the predictive power of accounting information on future firm development. Building on forecast relevance studies, valuation relevance studies analyze the predictive power of accounting information on equity or debt market variables and examine whether financial statement users consider deferred tax variables (Barth et al. 2001b; Beisland 2009).

#### Forecast relevance: income tax payments

In assessments of the forecast relevance of deferred taxes, the most apparent dependent variable is future income tax payments, as “a deferred tax liability or asset represents the increase or decrease in taxes payable or refundable in future years” (ASC 740-10-45-5). In predictions of future income tax payments using past values of income tax payments with and without net deferred taxes, the inclusion of deferred tax variables improves the predictive power of future income taxes up to five years ahead. This association is particularly significant in the first forecast year and is stronger for deferred tax assets than for deferred tax liabilities (Cheung et al. 1997; Jackson 2015; Murdoch et al. 2015; Johnston and Kutcher 2016). However, the timing of accounting and the specific components of deferred taxes (e.g., deferred tax assets from stock-based compensation) seem to limit this relation (Laux 2013; Johnston and Kutcher 2016), as only deferred tax assets and liabilities included in income before taxable income provide forward-looking information on future income tax payments for a forecast period of up to five years (Laux 2013).

As a result, depreciation-related deferred tax liabilities, which represent one of the largest items of deferred tax liabilities, are not forecast relevant, as assumed by ASC 740 (Laux 2013). This finding can, *inter alia*, explain the limited forecast relevance of deferred tax liabilities. Mear et al. (2019) criticize the low predictive ability of deferred taxes on future income tax payments. The authors find a forecast relevance of deferred taxes for future income taxes only if they exclude firm-year observations with losses. In the German setting, Chludek (2011) finds no relation between deferred tax expenses and future tax cash flows. This result may relate to the German accounting tradition in which firms did not focus on deferred tax expenses in early years (Chludek 2011) or indicate a lack of value relevance of deferred tax expense as recognized under IFRS.

#### Forecast relevance: cash flows/earnings

In contrast to future income tax payments, future cash flows/earnings depending on deferred taxes can provide a more comprehensive picture of firms’ future development. Early US studies based on SFAS No. 109 (ASC 740) find that changes in the valuation allowance, changes in deferred tax assets, and changes in deferred tax liabilities are associated with future firm profits (Cheung et al. 1997; Behn et al. 1998; Miller and Skinner 1998; Legoria and Sellers 2005). Recent studies emphasize that the valuation allowance, in particular, conveys private information about the firm’s future profitability (Edwards 2018; Dhaliwal et al. 2013). Significant increases in the valuation allowance precede significantly lower future earnings (Edwards 2018). Loss firm-years with material increases in the valuation allowance are more persistent (Dhaliwal et al. 2013). Comparing the predictive power of deferred taxes to that of total book-tax differences, Lev and Nissim (2004) find that total book-tax differences are more useful for predicting earnings changes than deferred taxes, as total book-tax differences capture deferred taxes, permanent differences, and tax accruals. Thus, earnings growth is positively associated with book-tax differences, although deferred taxes also provide information about future earnings changes up to five years ahead (Lev and Nissim 2004).

In contrast to total book-tax differences, earnings growth is negatively related to deferred tax expenses (Chi et al. 2014; Jackson 2015; Lev and Nissim 2004). Unlike Lev and Nissim (2004), Chi et al. (2014) do not find any significant relation between permanent book-tax differences and future earnings changes, and they conclude that only deferred taxes are indicative of future earnings changes.

The debate as to whether deferred taxes or permanent book-tax differences are incrementally useful to predict future earnings is ongoing. Hanlon (2005), Blaylock et al. (2012), and Jackson (2015) are examples attesting that deferred tax expenses have a higher predictive capacity for future earnings changes than do permanent book-tax differences.

Most of the results deal with periods after the implementation of SFAS No. 109 (ASC 740). The separate recognitions of deferred tax assets, deferred tax liabilities, and the valuation allowance implemented by the new standard increase the forecast relevance compared to the previously-required net amount of deferred taxes (Legoria and Sellers 2005). The early study of Beaver and Dukes (1972) supports this result, identifying only a weak correlation between net deferred taxes and future cash flows under APB No. 11.

The amount of deferred taxes limits the forecast relevance. The higher or lower deferred tax expenses are in comparison to other firms in the same jurisdiction, the lower the persistence of future earnings and the more difficult the prediction (Hanlon 2005; Zhou 2016). Wahab and Holland (2015) find substantial differences in the sector-specific accounting behavior of deferred taxes. In sum, deferred tax accounting under SFAS No. 109 (ASC 740) seems to meet the requirements of the liability view, with sector-specific differences.

Furthermore, an analysis must consider the country of origin and the field of activity of the firms. Comparing 33 different countries in terms of publicly-required book-tax conformity and earnings persistence, Atwood et al. (2010) find that the earnings persistence and the predictive potential for future cash flow are lower when the required level of book-tax conformity is higher. However, studies on non-US samples are rare. Studies from Australia, Canada, and Belgium show that deferred tax accounting conveys private information about the future profitability of (non-taxpaying) firms (Gaeremynck and Van De Gucht 2004; Herbohn et al. 2010). Herbohn et al. (2010) and Arcelus et al. (2005) find that the changes of deferred assets (liabilities) relate positively (negatively) to future performance variables (e.g., cash flow, EBITDA, ROA). In particular, the proportion and the change rate of recognized deferred tax assets from loss carryforwards convey private information about the future profitability of (non-taxpaying) firms, as this item is strongly correlated with future cash flows, EBITDA, and EBIT (Herbohn et al. 2010). Thus, studies outside the US also support the liability view and the forecast relevance of deferred taxes.

#### Valuation relevance: market value

A large portion of studies analyzes the relation between deferred taxes and the market value/share price of a firm. Overall, studies show that the capital market perceives deferred taxes—and especially deferred tax assets—as value relevant, and it includes them (at least partially) in market value and stock price (e.g., Chaney and Jeter 1994; Amir et al. 1997; Amir and Sougiannis 1999; Bauman and Das 2004; Lev and Nissim 2004; Chang et al. 2009; Samara 2014; Laux 2013; Marques et al. 2016). The accounting and valuation methods of SFAS No. 109 (ASC 740) bring about an improvement in value relevance compared to the previously enacted standard APB No. 11 (Ayers 1998). The ban on discounting deferred taxes leads to an overstatement of deferred tax liabilities in particular: regulators prohibit discounting, to facilitate deferred tax accounting and to reduce accounting costs. From a technical point of view, deferred taxes are discountable (Brouwer and Naarding 2018). Consistently, the market value depends on the retroactively-discounted deferred taxes, according to their approximated net present value (Amir et al. 1997). Deferred tax assets, in particular, convey information to the market, whereas the market perceives deferred tax liabilities as less value relevant (Ayers 1998; Chang et al. 2009; Wong et al. 2011; Bauman and Shaw 2016). Therefore, the market considers both recorded and unrecorded deferred tax assets (Lynn et al. 2008). Beyond these general results, the source of deferred tax assets (liabilities) is important for predicting their market valuation. For example, there is evidence that deferred tax liabilities on asset revaluations, as well as unrecorded and recorded deferred tax liabilities due to permanently-reinvested earnings, are value relevant (Bauman and Shaw 2008; Hanlon et al. 2014; McGuire al. 2016). Moreover, deferred tax assets resulting from tax loss carryforwards are value relevant (Zeng 2003).

Only a few studies do not find evidence for the valuation relevance of deferred tax assets (Citron 2001; Badenhorst and Ferreira 2016; Hanna et al. 2019) or deferred taxes in general (Chludek 2011). Citron (2001) shows that under former UK accounting rules, only deferred tax liabilities were value relevant, as financial statement users consider them for reversing. Other studies on UK firms present evidence that unrecognized and recognized deferred tax assets and liabilities provide value relevant information (Gordon and Joos 2004; Lynn et al. 2008). For Germany, Chludek (2011) finds no evidence for the market relevance of deferred taxes. This may be due to the national accounting tradition in Germany and to investors’ potential lack of experience in considering deferred taxes, even if they should report their income statements under IFRS as capital-market oriented German firms (Chludek 2011). Research on more recent periods is warranted to assess whether Chludek’s (2011) results hold for later periods when the use of IFRS is well established.

Badenhorst and Ferreira (2016) find a temporary lack of market relevance during the economic crisis of deferred tax assets for a cross-sectional sample of the largest Australian listed firms; in later years, the value relevance of deferred tax assets in the Australian capital market seems to decrease. Hanna et al. (2019) find that the market perception of banks’ net deferred tax assets was negatively affected by the economic crisis: market participants valued all components of banks’ net deferred tax assets more negatively than before the economic crisis. The general negative valuation of net deferred tax assets in the banking sector is caused by the divergent capital structure in comparison with industry firms. Still, the negative impact of the economic crisis on the value relevance of deferred taxes seems to be country-specific, as Badenhorst and Ferreira (2016) cannot reproduce their results with a cross-sectoral UK sample. Other crises, like the burst of the dot-com bubble, did not change the perception of the deferred tax assets of US internet firms (Bauman and Das 2004). These examples suggest obstacles to the value relevance of deferred taxes during an economic crisis.

#### Valuation relevance: abnormal returns

Beaver and Dukes (1972) present the first empirical results on the relation between abnormal returns and earnings and deferred taxes for the US capital market. They demonstrate that abnormal returns are more strongly correlated with unexpected after-tax results, including deferred taxes, than with results that only include current income taxes (Beaver and Dukes 1972). Investors seem to consider deferred tax liabilities as real liabilities, and they discount them in relation to the time and probability of realization before pricing into the firm value (Givoly and Hayn 1992; Amir et al. 1997). Evidence shows that almost all components of deferred taxes are (at least partly) recognized by the market (Amir et al. 1997; Amir and Sougiannis 1999; Comprix et al. 2011). For example, Amir et al. (1999), McGuire et al. (2016), and Herbohn et al. (2016) find that investors assign a positive value to (at least newly-generated) deferred tax assets from carryforward losses. An increase in deferred tax assets from carryforward losses is related to positive abnormal returns, especially if firms are about to miss the after-tax analyst forecasts, and thus seems to reveal a positive signal to investors (Herbohn et al. 2016). McGuire et al. (2016) provide results supporting the positive valuation of deferred tax assets from carryforward losses, especially if a firm is designated as a tax avoider.

Additionally, an announcement of changes in the valuation allowance or reported deferred tax adjustments conveys relevant information, which leads to changes in the abnormal stock returns (Kumar and Visvanathan 2003; Dhaliwal et al. 2013). This finding supports the signaling hypothesis. Sophisticated investors (e.g., short-seller, insider, and institutional investors) seem to exploit their understanding of the prediction capacity of deferred taxes to better predict future abnormal returns compared to small investors, who often misprice or ignore this information (Chi et al. 2014). The higher or lower the deferred taxes are, the less persistent the abnormal returns (Hanlon 2005). Furthermore, smaller numbers of analysts following a specific firm increase the probability of mispricing the deferred taxes of this firm (Chi et al. 2014). A widespread difficulty is the phenomenon of IPO underperformance compared to the market; many firms fail or disappoint investors with negative abnormal returns during the first years. Studying the cumulative abnormal returns and abnormal buy-and-hold returns of newly public firms, a recent study of the German capital market finds that net deferred tax assets are strongly predictive of superior post-IPO long-run performance (Kovermann and Velte 2019).

#### Valuation relevance: analysts’ forecasts

Since investors use analysts’ earnings forecasts as a key source of information (Amir and Sougiannis 1999), it is necessary to understand how analysts incorporate deferred taxes into their forecasts. Empirical evidence suggests that analysts correctly value the information inherent in deferred tax variables (Comprix et al. 2011; Chi et al. 2014). Analysts consider single components of deferred tax expenses, with a special focus on carryforward losses. The existence of deferred tax assets from carryforward losses generally indicates less persistent earnings in the future caused by the increased likelihood of future losses; thus, analysts expect that firms without carryforward losses will generate higher future incomes. Moreover, the evaluation of deferred tax expenses from carryforward losses is related to the disclosed valuation allowance: the smaller the valuation allowance, the higher the likelihood that the carryforward losses can be used in the future. This translates into higher forecasted earnings as compared to firms with a large valuation allowance (Amir and Sougiannis 1999). Other studies motivated by the Omnibus Budget Reconciliation Act in 1993, which caused corporate income tax to increase by 1%, indicate the correct interpretation of deferred tax liabilities changes caused by this one-time tax effect (Chen and Schoderbek 2000; Chen et al. 2003). The studies examine whether analysts mechanically react to changes in deferred tax liabilities caused by the Omnibus Budget Reconciliation Act without understanding the effect source. While studies show that analysts have ignored these changes, it can be assumed that analyst forecasts only include deferred tax expenses if they can impact the future firm value (Chen and Schoderbek 2000; Chen et al. 2003).

#### Valuation relevance: credit rating

In contrast to studies that assess the equity market, studies assessing the debt market cannot directly examine creditors’ interpretation of deferred taxes, as this information is not publicly available. Two recent studies analyze the association between the cost of debt and deferred tax expenses, finding that the debt market interprets increased deferred tax expenses as a signal of low earnings quality, which results in higher costs of private debt (Inamura and Okuda 2017; Moore and Xu 2018). Inamura and Okuda (2017) find a significant negative relation among creditworthiness—measured by, e.g., the credit spread of Japanese public—nonconvertible debt contracts, and unusually large deferred tax expenses. Other studies analyze the relation between credit ratings or credit spread and deferred taxes to assess the debt market’s perception of deferred taxes. These studies find a negative relation between assigned bond ratings, large positive deferred taxes, and increases in the valuation allowance (e.g., Crabtree and Maher 2009; Edwards 2018).

The initial impetus for US scholars to assess bond and credit ratings was high-profile accounting scandals (Enron, WorldCom, Xerox) at the beginning of the twenty-first century. The misstatements have motivated creditors and analysts to focus more on the quality and the components of balance sheet information. Deferred taxes may convey relevant information for credit rating agencies. First, notable high or low deferred tax expenses signal lower earnings quality (Hanlon 2005). In particular, increasing book-tax differences preceded the accounting scandals. Second, large deferred tax assets can signal off-balance-sheet financing, which conveys information about the creditworthiness of a firm (Crabtree and Maher 2009). For the period after the Enron scandal, Crabtree and Maher (2009) find a U-shaped relation between bond ratings and deferred taxes, which reflects the negative correlation between deferred tax assets (liabilities) in the highest (lowest) quantile and rating changes (Crabtree and Maher 2009). Moreover, Edwards (2018) observes a negative relation between increases in the valuation allowance and credit ratings. Only an early study on Canadian firms does not find a relation between deferred tax variables and credit ratings (Chattopadhyay et al. 1997), but their sample predates the major accounting scandals. These results confirm Crabtree and Maher’s (2009) assumption that rating agencies have only reacted sensitively to deferred taxes in the wake of the accounting scandals.

#### Summary of the results from the narrative synthesis of the value relevance studies

The vast majority of value relevance studies show that deferred taxes are forecast relevant to predicting future income tax payments (e.g., Cheung et al. 1997; Laux 2013; Murdoch et al. 2015). Furthermore, the majority of studies find evidence of the predictive capacity of deferred taxes for future cash flows and earnings changes (e.g., Hanlon 2005; Dhaliwal et al. 2013; Edwards 2018). In both cases, deferred tax assets and the valuation allowance are perceived as more value relevant than deferred tax liabilities. In some studies, researchers presume that deferred tax assets are the value relevant variable and thus exclude deferred tax liabilities (e.g., Bauman and Das 2004; Badenhorst and Ferreira 2016). However, the exclusion of deferred tax liabilities potentially biases research results toward overestimating the value relevance of deferred tax assets.

The capital market perceives deferred taxes as value relevant. The size of deferred tax variables, along with changes in deferred tax variables, are useful for predicting future market values and abnormal returns (e.g., Chaney and Jeter 1994; Amir et al. 1997; Amir and Sougiannis 1999; Bauman and Das 2004; Lev and Nissim 2004; Chang et al. 2009). Critics argue that the prohibition on discounting deferred tax assets and liabilities leads to an overstatement of deferred taxes, which reduces the value relevance of deferred taxes. The mandatory capitalization of deferred tax liabilities combined with the prohibition on discounting deferred tax liabilities causes the market to perceive deferred tax liabilities as less value relevant (Ayers 1998; Chang et al. 2009; Wong et al. 2011; Bauman and Shaw 2016). However, Amir et al. (1997) show that the market valuation depends on the retroactively-discounted deferred taxes.

Because of the value relevance of deferred taxes, analysts, rating agencies, and credit providers use the information inherent in deferred taxes when evaluating a firm (e.g., Crabtree and Maher 2009; Comprix et al. 2011; Chi et al. 2014; Inamura and Okuda 2017; Edwards 2018).

### Narrative synthesis of the earnings management studies

Our categorization framework classifies earnings management research according to the earnings management measures, leading to categorizations of studies that use mis-/re-statements, thresholds, and accruals. Such research analyzes whether deferred taxes are an instrument for or an indicator of earnings management.

#### Earnings management: mis-/re-statements

Earnings management often precedes mis-/re-statements, and the analysis of mis-/re-statements provides insights on whether deferred taxes can identify earnings management. Ettredge et al. (2008) show that deferred tax expenses increase in the year before fraudulent accounting, compared to a set of control firms without mis-/re-statements. In the fraud year, the observed deferred tax expenses are further increased, which indicates that an increase in deferred tax expenses (compared to non-fraudulent firms) can signal earnings management and accounting fraud. Ettredge et al. (2008) include only firms with upward earnings management and cluster them depending on the amount of deferred tax expenses. The authors exclude firms that operate in the utilities and financial services sectors, as well as mis-/re-statements detected only in quarterly income statements and all misstatements before 1988. Dechow et al. (2011) use similar data without these restrictions, and they fail to reproduce the findings in Ettredge et al. (2008). Thus, deferred tax expenses do not seem to be a general indicator for earnings management, even though they can identify upward earnings management. Badertscher et al. (2009) investigate whether deferred taxes, especially deferred tax assets from carryforward losses, are useful in identifying upward earnings management. They conclude that deferred taxes and particularly large deferred tax assets from carryforward losses are associated with upward earnings management (Badertscher et al. 2009).

Further studies for several countries show that proposed audit adjustments are positively related to large book-tax differences. These studies find that deferred tax expenses are a better predictor for upward earnings management than book-tax differences (Mills 1998; Cho et al. 2006; Wilson 2009; Badertscher et al. 2009). As a result, positive proposed audit adjustments, which increase taxable income, are most studied. This focus is partly due to the intrinsic characteristics of audit adjustments (Mills 1998; Cho et al. 2006). Consequently, increased deferred taxes can indicate upward earnings management.

#### Earnings management: thresholds

The motivation for a firm to manage earnings increases if a certain accounting number is just below the target earnings threshold (Healy and Wahlen 1999). Drawing on thresholds, such as prior year earnings or analyst forecasts, studies can examine whether a firm’s managed earnings meet or beat a certain threshold. The studies discussed in this section use this instrument to analyze whether deferred taxes are an instrument for earnings management or whether they can identify earnings management. To be sure, thresholds are the most popular measure in this field of research. While investigating whether deferred taxes are appropriate for identifying earnings management, Phillips et al. (2003, 2004) use thresholds as a benchmark for earnings management against earnings decline (Phillips et al. 2003, 2004). They put forth the hypothesis that managers have more discretion under US GAAP than under tax rules to manage earnings, and if they exploit their discretion (e.g., to manage earnings upwards), deferred taxes arise because of earnings management.

To classify a firm as an earnings manager to avoid earnings decline, Phillips et al. (2003, 2004) calculate the change in a firm’s net income divided by the market value of equity. If this ratio is greater than zero but smaller than 0.1, a firm is classified as an earnings manager. The authors find that deferred tax expenses are more appropriate than accruals for identifying upward earnings management. Phillips et al. (2003) find no evidence that deferred tax expenses can identify earnings management to meet analyst forecasts. In a subsequent study using a subsample of Phillips et al. (2003, 2004) decompose the total deferred tax amount into several components—e.g., revenue and expense accruals and reserves, depreciation- or compensation-related deferred taxes, and the valuation allowance. They analyze which of these components is incrementally useful in identifying upward earnings management and find that the previously identified relation between deferred tax expenses and upward earnings management still hold for all components. The relation is especially strong for the deferred tax component reflecting revenue and expense accruals and reserves (Phillips et al. 2004).

Further US-based studies find significant associations between increased deferred tax expenses and upward earnings management (Dhaliwal et al. 2004; Zhou 2016). Dhaliwal et al. (2004) investigate the case of upward earnings management to meet analyst forecasts while reducing the fourth quarter effective tax rate. They find that firms that miss the forecast in the third quarter are more likely to decrease the effective tax rates and have higher deferred tax expenses compared to firms that beat the forecast. In contrast, Jackson (2015) is not able to reproduce the results of Phillips et al. (2003). Jackson (2015) investigates a large cross-sectoral US sample. This leads to the assumption that the results from Phillips et al. (2003) seem sensitive to changes in the research design (e.g., different threshold calculations to identify a firm as an earnings management, different sample restrictions).

Other studies use thresholds to assess whether deferred taxes are an instrument to manage earnings. Under US GAAP, the valuation allowance involves a large margin of discretion, which can be used opportunistically. Early studies find that the valuation allowance is set according to the economic circumstances (Behn et al. 1998; Miller and Skinner 1998; Kumar and Visvanathan 2003; Phillips et al. 2004; Chao et al. 2004).

Further studies exploit specific regulatory settings for banks. Using a small sample of commercial US banks, Schrand and Wong (2003) are the first to find that the valuation allowance is used to smooth earnings while building hidden reserves, the so-called ‘cookie jar reserves’. In the course of the introduction of SFAS 109, banks could recognize high amounts of deferred tax assets and had the opportunity to build these reserves. However, due to the small sample size and the one-time effect caused by the significant legal change, the results of Schrand and Wong (2003) are far from generalizable. Skinner (2008) investigates a setting during the Japanese banking crisis wherein weak banks reduced their valuation allowances below an amount that is economically necessary to reach the regulatory capital. A significant minority of banks only reached the regulatory capital by including deferred tax assets. The full inclusion of deferred tax assets into the regulatory capital is only allowed in few countries, as is the special incentive to manage earnings (Skinner 2008). Junqueira and Nakao (2013) confirm the results of Skinner (2008) for Brazilian banks.

The first cross-sectional evidence for the opportunistic use of valuation allowance is provided by Frank and Rego (2006). They examine a large sample of US firms and present evidence consistent with managers using the valuation allowance to meet analyst forecasts and to manage earnings upwards, but they do not find evidence of earnings management through accounting for other earnings targets (e.g., big bath accounting, building cookie jar reserves). These findings are confirmed by Christensen et al. (2008), who do not find indications that the valuation allowance is used to balance a big bath or cookie jar reserves. However, they do provide evidence that the valuation allowance is used to meet or beat analyst forecasts (Christensen et al. 2008). In the Malaysian setting, Kasipillai and Mahenthiran (2013) find evidence for the opportunistic use of the valuation allowance: in particular, concentrated ownership and small boards of directors increase the likelihood of earnings management activities (Kasipillai and Mahenthiran 2013).

Moreover, several studies with US, Australian, Malaysian, Hong Kong, and UK firms indicate that deferred tax expenses are potentially used to reach analyst forecasts and to manage earnings upwards (Gordon and Joos 2004; Holland and Jackson 2004; Herbohn et al. 2010, 2016; Richardson and Leung 2011; Kasipillai and Mahenthiran 2013). The Australian studies also find that the percentage of recognized deferred tax assets from carryforward losses increases if the pre-tax income is below the forecasted one. This finding is interpreted as signaling earnings management. However, the findings do not invalidate the predictive power of the recognition rate of deferred tax assets on future cash flows. Consequently, it is uncertain whether the identified earnings management activities are economically significant (Herbohn et al. 2010, 2016). In contrast, using a limited US sample with strong incentives for earnings management, Stammerjohan and Hall (2003) show that deferred tax expenses are set exclusively according to economic circumstances (Stammerjohan and Hall 2003).

#### Earnings management: accruals

A final research strand uses accruals to identify and measure earnings management and primarily addresses the relations between earnings management, earnings quality, and deferred taxes. These studies use either total accruals (with no distinction between normal and discretionary accruals) or the modified Jones model to measure discretionary accruals. It is worth noting that conceptually, only discretionary accruals are assumed to relate to earnings management (Jones 1991; Healy and Wahlen 1999).

Phillips et al. (2004) find that deferred tax expenses are a better predictor of earnings management than accruals are. Deferred tax expenses are particularly more useful in detecting earnings management for firms that avoid earnings decline. Hanlon et al. (2012) analyze whether high audit fees are related to large book-tax differences and large deferred taxes; they find that the auditors interpret both large book-tax differences and large deferred tax expenses as a potential risk factor for earnings management, which results in higher audit fees. In this way, Hanlon et al. (2012) identify earnings management firms based on total accruals. Several studies using the modified Jones model find that upward earnings management is related to increased deferred tax expenses, reduced disclosure transparency concerning the deferred tax-related valuation allowance, and less persistent future earnings (Blaylock et al. 2012; Cassell et al. 2015; Kapoutsou et al. 2015). Only Jackson (2015) and Dechow et al. (2011) find no clear relation between earnings management and deferred taxes through the use of accruals to identify earnings management firms. Overall, five out of seven studies suggest a relation between upward earnings management and deferred tax expenses; however, the results seem to be sensitive to the accruals measure used, as well as other aspects of the research design.

#### Summary of the results from the narrative synthesis of the earnings management studies

Earnings management studies based on mis-/re-statements suggest that unusually large (low) deferred tax expenses are related to earnings management. They show that firms that manage earnings upwards (tax downwards) have particularly large deferred tax expenses (e.g., Cho et al. 2006; Ettredge et al. 2008; Badertscher et al. 2009). In a way, these studies bridge the gap between value relevance studies and earnings management studies.

In a comparison of the predictive capacity of deferred tax expenses, specific thresholds, and accruals to identify earnings management, more than two-thirds of the included studies found evidence that deferred tax expenses are at least as indicative as accruals or thresholds (e.g., Phillips et al. 2003, 2004; Blaylock et al. 2012; Zhou 2016). The results are valid for the earnings management motivator of upward earnings management but do not present evidence of the identification of earnings management through deferred taxes for any other earnings target (e.g., Jackson 2015).

Specific earnings management thresholds are also used to assess whether deferred tax expenses are an instrument of opportunistic accounting. Several studies show a relation between earnings management and increased deferred tax expenses (e.g., Herbohn et al. 2010; Richardson and Leung 2011; Kasipillai and Mahenthiran 2013). These studies identify a significant relation only between earnings management and deferred tax expenses; they do not identify the discretion used to influence earnings opportunistically with deferred taxes. Based on these results, a distinction as to whether deferred tax expenses are an instrument or a result of earnings management is not possible. As to studies examining the margin of discretion to determine the amount of the valuation allowance—which represents the most significant influencing factor in managing earnings with deferred tax assets—the evidence is mixed (e.g., Phillips et al. 2004; Frank and Rego 2006; Skinner 2008). The valuation allowance may be an instrument to manage earnings in some cases, but the combined research results do not indicate a systematic opportunistic use of the valuation allowance and deferred taxes for earnings management.

## Implications for future research

Our literature review has a number of implications for future research on financial accounting for deferred taxes, which we deem to be a fertile field for empirical research. In this section, we discuss three methodological implications and six content-driven implications, thereby suggesting avenues for future research.

### Methodological implications

Our bibliographic analysis indicates that the body of empirical studies has substantially grown in the last decade (2010–2019). First, this trend will offer opportunities for meta-analytic methods to address the aggregate effects of deferred taxes (and moderating factors). For example, our review identifies 42 studies on the valuation relevance of deferred taxes on equity markets. As the number of such studies increases, researchers can meta-analytically assess the associations between market variables and deferred tax items.

Second, most of the value relevance studies (38) and some of the earnings management studies (4) reviewed refer to the Ohlson (1995) or the Feltham and Ohlson (1995) models. Both models assume that stock prices are a linear function of earnings (abnormal earnings) and the book value of equity. Literature questions the assumption of a linear function in value relevance studies (Barth et al. 2001b; Holthausen and Watts 2001). Thus, alternative models to examine the value relevance of deferred taxes will be warranted. Alternatives include, e.g., ordered logit models with additional control variables (Edwards 2018), and non-linear residual income models that explicitly consider growth opportunities (Biddle et al. 2001).

Third, the majority of value relevance studies reviewed focuses either on the combined variable net deferred taxes (the difference between deferred tax assets and deferred tax liabilities) or on the variables deferred tax assets and liabilities. As deferred tax assets and liabilities reflect an aggregate of components arising from various sources and business transactions, the analysis of the individual components of these variables can provide more differentiated insights. Recent studies have shown that the value relevance of deferred taxes is related to the sources of temporary differences (Laux 2013; Johnston and Kutcher 2016; Hanna et al. 2019). However, only 13 studies reviewed separately analyze deferred tax variables according to their underlying sources. This limitation suggests substantial room to contribute to a finer graded our understanding of the drivers of the value relevance of deferred taxes.

### Content-driven implications

Our bibliographic analysis reveals that empirical research has focused on the US setting and financial accounting under US GAAP. First, the focus on the US setting suggests room for future research to exploit particularities of other national settings, for example, in course of the implementation of new financial or tax accounting regulations and code law settings in general, and to conduct cross-country studies.

Second and relatedly, it is surprising how little attention empirical research has devoted to deferred taxes under IFRS. Only 12% of the studies reviewed are based on firms applying IFRS. Samples of IFRS firms, however, offer various avenues for future research in both strands of research discussed in this review. Most notably, international samples of IFRS users enable investigating how country-level, institutional or cultural frameworks relate to value relevance and firms’ use of deferred taxes to manage their earnings while keeping the financial accounting regulations stable. Moreover, the adoption of IFRS by firms seems worth examining since the first time adoption of IFRS offers substantial room for managerial discretion for financial accounting for deferred tax under the provisions of IFRS 1. Such research will be warranted to inform the ongoing debate on accounting for deferred taxes under IFRS at the IASB (e.g., IASB 2017, 2020).

Third, the narrative synthesis of empirical evidence indicates that various factors investigated in general financial accounting research on value relevance and earnings management have been neglected in studies on deferred taxes. For example, financial accounting research suggests an association between corporate governance and both, value relevance and earnings management. Related evidence on deferred taxes, however, remains scarce. Notably, Kasipillai and Mahenthiran (2013) find that board structure and ownership structure are related to the extent to which earnings management is associated with deferred taxes in Malaysia. More research into aspects of corporate governance is important to understand how value relevance of and earnings management through deferred taxes are affected by firm-level institutional factors. As financial accounting and tax literature indicate, the list of such institutional factors is extensive and multifaceted. Examples include board diversity, executive compensation, family ownership, financial auditor characteristics, firm risk, legal form, listing status, and tax auditor characteristics.

Fourth, some studies reviewed have taken advantage of specific settings in the banking sector (e.g., Skinner 2008; Hanna et al. 2019). We assume that there are other, highly regulated sectors, offering unique settings to particularly assess how firms use deferred taxes to manage their earnings. For example, insurance firms can have incentives to use deferred tax assets to reach regulatory capital thresholds if the inclusion of deferred tax assets in regulatory capital is allowed. More generally, larger deferred tax assets ceteris paribus reduce the debt ratio, which can have positive effects on the assessments of firm risk and creditworthiness. Based on this relation, it would be interesting to assess whether firms in predominantly debt-financed sectors are more inclined to use managerial judgment to increase deferred tax assets vis-à-vis firms in other sectors. Single-sector settings also seem to be suited to distinguish between earnings management that is directly related to discretion in deferred tax accounting and earnings management that indirectly affects the amounts of deferred tax items.

Fifth, the increasing role of corporate social responsibility (CSR) triggers empirical research on the association between CSR and corporate tax avoidance. This emerging strand of research yields mixed results to date by relying on total, discretionary, or permanent book-tax differences as a measure for tax avoidance (Kovermann and Velte 2021). Thus, it would be interesting to incorporate deferred taxes. For example, future research of this strand could exploit the finding of Blaylock et al. (2012) and Jackson (2015) that deferred taxes serve as a signal for earnings management and tax avoidance.

Finally, the Covid-19 pandemic offers abundant settings to contribute to financial accounting research for deferred taxes by investigating the effect of the economic shock and changes in tax regulations. Since our review reveals mixed results on the value relevance of deferred taxes during economic crises (Bauman and Das 2004; Badenhorst and Ferreira 2016), further evidence will be warranted to better understand financial statement users’ perception of deferred tax items, deferred tax assets in particular, in times of crisis. As the economic shock caused by the Covid-19 pandemic causes high cross-sectoral losses, firms are likely to be inclined to manage earnings upwards. The losses lead to an increase of the discretionary deferred tax component of loss carryforwards, a component that firms are likely to use to manage their earnings as shown in our review. It will thus be interesting to investigate whether deferred tax assets from tax carryforwards increase and whether an increase is reflected in market values. Results will be of interest because governments around the world support the recognition of long-term tax loss carryforwards (e.g., Deloitte 2020; KPMG 2020). Around the world, there are many other changes in tax regulations and tax relief measures taken to support firms during the Covid-19 pandemic (IRD 2021; KPMG 2021) that affect deferred taxes and offer opportunities for studies on value relevance, earnings management, or both.

## Conclusion

This review was motivated by the contentious debates on deferred tax accounting concepts and standards around the world (Brouwer and Naarding 2018; Morton 2019; IASB 2018), the inconclusive and contradictory empirical research results (Phillips et al. 2003; Jackson 2015), and the different treatments of deferred tax variables in recent research (Krishnan and Visvanathan 2011; Huang and Chang 2016; Kraft and Lopatta 2016; Graham and Moore 2018). To provide a more comprehensive understanding of deferred tax accounting, our paper reviewed the prevailing empirical literature in the areas of value relevance and earnings management, taking both a bibliographic and a content-driven perspective.

The bibliographic analysis reveals that the number of empirical research articles on deferred tax accounting has increased over time. A total of 76 high-profile empirical studies on accounting for deferred taxes that meet the requirements of this review were published in the last three decades. In the first decade (1990–1999), empirical research is focused on value relevance. In the second and third decades (2000–2009; 2010–2019), studies analyze topics related to both earnings management and value relevance. Most studies examine one topic and focus on one country; cross-topic and cross-country studies are rare. Throughout the three decades, the proportion of US studies is predominant; however, the share of non-US studies has been growing, especially in the third decade (2010–2019). This latter finding suggests that empirical research in the field has just begun to go beyond investigating the US setting, where regulations on financial accounting for deferred taxes have their origins.

The narrative synthesis of the value relevance research reveals that deferred tax assets and deferred tax expenses are value relevant in predicting future income taxes, cash flows, earnings, market values, and abnormal returns, as well as rating changes and future credit risks. The results concerning the value relevance of deferred tax liabilities vary. Overall, the results indicate that deferred taxes are informative for financial statement users in terms of value relevance.

The information content of deferred taxes goes beyond the prediction of financial statements and market values, as the narrative synthesis of the earnings management studies shows that deferred tax variables—especially deferred tax assets—are an indicator of upward earnings management. The results of the earnings management studies do not indicate that deferred tax variables are systematically used for earnings management. However, research demonstrates that in specific settings, firms use deferred taxes for earnings management purposes.

Cross-topic studies show that financial statement users (such as investors and auditors) interpret large positive and large negative deferred tax expenses, along with large deferred tax assets, as a “red flag” and consequently reduce their expectations of future earnings and market value development. This leads to the conclusion that the value relevance of deferred taxes is limited by their potential to identify earnings management.

We suggest several avenues for future research. Those avenues include more and more detailed empirical work on deferred taxes under IFRS. From a regulatory point of view, research on deferred taxes under IFRS is warranted to inform the ongoing debate at the IASB. In 2017, the IASB added the research topic “deferred taxes” to its current work plan of “better communication in financial reporting” (IASB 2017). This addition was rather surprising because in 2016 the IASB decided not to change IAS 12 (IASB 2016c). While our review is largely based on US samples, it may provide some indications for this regulatory debate. For example, the content-driven analysis of the value relevance literature indicates that deferred tax liabilities are less value relevant than deferred tax assets. The prohibition on discounting deferred tax items and the mandatory capitalization of deferred tax liabilities for all temporary differences leads to a significant overstatement of deferred tax liabilities, which reduces its value relevance. In contrast to deferred tax liabilities, deferred tax assets can be value-adjusted if necessary, and their capitalization depends on probable future taxable income. As a result, value relevance literature reveals that deferred tax assets provide decision-useful information, while deferred tax liabilities often do not. Moreover, the current review may contribute to the ongoing debate regarding the IASB project “Deferred Tax related to Assets and Liabilities arising from a Single Transaction (Amendments to IAS 12)” (IASB 2020). On the one hand, our review shows that unrecognized deferred tax assets and liabilities provide value relevant information, which seems to support the mandatory recognition of deferred tax related to assets and liabilities arising from a single transaction (Gordon and Joos 2004; Lynn et al. 2008). On the other hand, only deferred tax assets and liabilities included in GAAP income before taxable income provide decision-useful information (Laux 2013). Given that leases and decommissioning result in deferred taxes recognized first in taxable income, it is uncertain whether the amendments provide additional decision-useful information.

Our study is not free of limitations. First, our study only reviews empirical research articles; the inclusion of analytical and qualitative articles may have revealed further insights and enhanced the discussion of the results. Second, the presented results and the selection of articles follow the rigorous approach of Tranfield et al. (2003). Notably, we excluded empirical studies that focus on issues of value relevance and earnings management related to financial accounting for deferred taxes. Future studies can broaden or amend the scope by including, for example, empirical studies that are more focused on tax accounting or that come from other adjacent disciplines. Finally, we excluded papers published in low-ranked journals, books, working papers, and ‘gray literature’; such forms of literature may nevertheless contain interesting findings and offer additional insights.

## Appendix

See Table

**Table 4:** Overview of empirical deferred tax accounting literature—a thematical list of articles included in the review

Article	Country and GAAP	Sample size	Sample period	Deferred tax variable	Main results	Contribution to key issues				
						A	B	C	D	E
*Primary research area: value relevance (contribution to key issues A and B)*										
Mear et al. (2019)	New Zealand GAAP, IFRS	1254 firm-year obs	1999–2013	Total DTE, DTA, DTL	Deferred taxes increase the ability to predict future taxes, especially while excluding loss-making firm-years	✓	–	–	–	–
Johnston and Kutcher (2016)	US GAAP	2432 firm-year obs	2006–2012	Components DTA	Components of DTA provide incremental information about future tax payments	✓	–	–	–	–
Atwood et al. (2010)	Local GAAP (33 countries)	93,893 firm-year obs	1992–2005	Total/change rate/components DTE	The higher or lower DTE are, the lower are earnings persistence and the association between current and future earnings and cashflows	✓	–	–	–	–
Legoria and Sellers (2005)	US GAAP	1642 firms	1994–1998	Total DTA/DTL, VA	DTA/DTL and VA provide forward-looking information to predict future cashflows	✓	–	–	–	–
Gaeremynck and Van De Gucht (2004)	Belgium GAAP	628 firms	1991–1995	Components DTA, DTL	The recognition and timing of deferred tax liabilities conveys private information about the future profitability	✓	–	–	–	–
Miller and Skinner (1998)	US GAAP	200 firms	1992–1993	Total DTA, VA	The VA is set in accordance with the economic circumstances of the firms, and legal requirements of SFAS 107	✓	–	–	–	–
Cheung et al. (1997)	US GAAP	51,519 firm-year obs	1973–1990	Total DTE	Total DTE enhance the prediction of future cashflows and tax payments	✓	–	–	–	–
Hanna et al. (2019)	US GAAP	433 banks	2006–2010	Total net DTA, components DTA	During the financial crisis, total net DTA and components of DTA have a negative relation to stock prices	–	✓	–	–	–
Kovermann and Velte (2019)	IFRS	80 firm-year obs	2005–2015	Total net DTA	Total net DTA in the year before IPO are positively related to the post-IPO performance of a firm	–	✓	–	–	–
Edwards (2018)	US GAAP	9432 firm-year obs	1993–2015	Total VA	The VA for DTA is positively related to the future creditworthiness of a firm	–	✓	–	–	–
Moore and Xu (2018)	US GAAP	6336 firm-year obs	1996–2012	Total DTE	DTE are positively related to the costs of private debt	–	✓	–	–	–
Inamura and Okuda (2017)	Japanese GAAP	890 debt contracts	2001–2006	Total DTA/DTL, VA	DTL are positively related to the costs of private debt whereas DTA are not. Investors evaluate components of deferred taxes differentiated	–	✓	–	–	–
Badenhorst and Ferreira (2016)	IFRS	848 Australian and 1038 UK firm-year obs	2005–2011	Total DTA	During the financial crisis, the value relevance of DTA was reduced for the Australian sample and remain unaffected for the UK sample	–	✓	–	–	–
Bauman and Shaw (2016)	US GAAP	10,389 firm-year obs	1994–2014	Net DTA/DTL, current/non-current, DTA/DTL	The distinction between current and non-current DTA/DTL provides value relevant information	–	✓	–	–	–
Marques et al. (2016)	IFRS	5065 firm-year obs	2002–2013	Total DTE	Total DTE are negatively related to EPS	–	✓	–	–	–
McGuire et al. (2016)	US GAAP	7010 firm-year obs	1993–2012	Total DTA, VA	Only newly recognized VA are positively related to the market value	–	✓	–	–	–
Murdoch et al. (2015)	US GAAP	4700 firm-year obs	1994–2013	Total DTE	Total DTE are predictive for future tax expenses, especially for shorter (one or two year) forecast horizons	–	✓	–	–	–
Wahab and Holland (2015)	IFRS	798 firm-year obs	2005–2010	Total DTE	Total DTE are impersistent what indicates a non-earnings management motivation	–	✓	–	–	–
Hanlon et al. (2014)	Australian GAAP	291 firms	2005–2006	Net DTA/DTL, components DTL	Deferred tax balances provide value relevant information. Components of DTL are real liabilities	–	✓	–	–	–
Samara (2014)	IFRS	1200 firm-year obs	2005–2013	Net DTA/DTL	Net DTA/DTL are negatively related to the market value of equity	–	✓	–	–	–
Comprix et al. (2011)	US GAAP	9687 firm-year obs	1991–2008	Total DTE	DTE are positively related to share turnover, analyst forecast and stock return variance	–	✓	–	–	–
Wong et al. (2011)	IFRS/New Zealand GAAP	411 firm-year obs	2000–2004	Disclosed/non-disclosed DTL	DTL recognized under IFRS are not value relevant whereas DTL recognized under the partial method are value relevant	–	✓	–	–	–
Chang et al. (2009)	Australian GAAP	478 firm-year obs	2002–2004	Total/change rate DTA/DTL	DTA/DTL recognized under the income statement method are viewed as assets and liabilities	–	✓	–	–	–
Crabtree and Maher (2009)	US GAAP	1843 firm-year obs	1994–2004	Total DTE	Large negative or large positive DTE are negatively related to bond ratings (U-shaped relation)	–	✓	–	–	–
Bauman and Shaw (2008)	US GAAP	835 firm-year obs	2000–2004	Components DTE	Footnote disclosure regarding DTE from untaxed foreign earnings are value relevant	–	✓	–	–	–
Lynn et al. (2008)	UK GAAP	1628 firm-year obs	1993–1998	Recognized/non-recognized DTA	Recognized and non-recognized DTA are positively related to the share price of a firm	–	✓	–	–	–
Arcelus et al. (2005)	Canadian GAAP	1877 firm-year obs	1990–1999	Total DTE	DTE are negatively related to the market value	–	✓	–	–	–
Bauman and Das (2004)	US GAAP	176 firm-year obs	1999–2000	Total DTA/DTL, components	DTA from internet firms are positively related to stock prices before and after the market correction in 2000 (dot-com bubble)	–	✓	–	–	–
Gordon and Joos (2004)	UK GAAP	3912 firm-year obs	1993–1998	Total DTA/DTL, unrecognized DTA/DTL	Unrecognized and recognized DTA/DTL provide value relevant information to investors	–	✓	–	–	–
Chen et al. (2003)	US GAAP	114 firms	1993	Net DTA/DTL	Analysts value net DTA/DTL adjustments according to their actual impact on the market value	–	✓	–	–	–
Zeng (2003)	Canadian GAAP	359 firms	1996–1998	Total DTA, components DTA	DTA from loss carryforwards provide value relevant information	–	✓	–	–	–
f	UK GAAP	1736 firm-year obs	1989–1991	Total DTA/DTL, disclosure	DTL provide value relevant information. DTA do not provide value relevant information to the market participants	–	✓	–	–	–
Chen and Schoderbek (2000)	US GAAP	158 tax adjustments	1993	Total/change rate DTA/DTL	Analysts and investors value net DTA/DTL adjustments according to their actual impact on the market value	–	✓	–	–	–
Amir and Sougiannis (1999)	US GAAP	1058 firm-year obs	1992–1995	Total DTA/DTL, components DTA, VA	Analysts rate earnings of firms with loss carryforwards as less sustainable. DTA and DTA from loss carryforwards are value relevant for investors	–	✓	–	–	–
Ayers (1998)	US GAAP	988 firms	1992–1993	Total DTA/DTL, VA	The separate disclosure of DTA and DTL is value relevant	–	✓	–	–	–
Amir et al. (1997)	US GAAP	1114 firm-year obs	1992–1995	Total DTA/DTL	DTA/DTL and DTA from loss carryforwards are value relevant	–	✓	–	–	–
Chattopadhyay et al. (1997)	Canadian GAAP	79 firm-year obs	1973–1990	Net DTA/DTL	No relation between net DTA/DTL and credit ratings	–	✓	–	–	–
Chaney and Jeter (1994)	US GAAP	14,242 firm-year obs	1969–1985	Total DTE	Total DTE are negatively related to the market value	–	✓	–	–	–
Givoly and Hayn (1992)	US GAAP	1348 firm-year obs	1986	Total/Change rate DTL	Investors interpret DTL as liability and discount these as a function of their respective lifetimes	–	✓	–	–	–
Chi et al. (2014)	US GAAP	78,320 firm-year obs	1988–2009	Total DTE	DTE reflect firm’s future profitability. Sophisticated/institutional investors better interpret information inherent in DTE than investors in general	✓	✓	–	–	–
Dhaliwal et al. (2013)	US GAAP	21,912 firm-year obs	1993–2008	Change rate DTA, VA	The VA provides information about the persistence of losses. The valuation of the VA depends on the environment of the firm	✓	✓	–	–	–
Huang and Wang (2013)	Taiwan GAAP	214 firm-year obs	1996–2006	Total DTE	Large positive or large negative DTE are related to loan loss provisions that are greater than for bank-years with average DTE	✓	✓	–	–	–
Laux (2013)	US GAAP	2763 firm-year obs	1994–2007	Components DTA/DTL	DTA/DTL that are included in GAAP income prior to taxable income are related to future income tax payments and market prices	✓	✓	–	–	–
Chludek (2011)	IFRS	626 firm-year obs	2005–2008	Total DTA/DTL, VA	DTA/DTL nor VA are value relevant. They do not provide information about future cashflows or market values	✓	✓	–	–	–
Hanlon (2005)	US GAAP	14,106 firm-year obs	1994–2000	Total DTE	Large positive or negative DTE are related to lower earnings persistence and act as a "red flag" for investors	✓	✓	–	–	–
Lev and Nissim (2004)	US GAAP	40,372 firm-year obs	1973–2000	Net DTA/DTL	Net DTA/DTL are value relevant to predict future earnings and stock returns	✓	✓	–	–	–
Beaver and Dukes (1972)	US GAAP	123 firms	1963–1967	Net DTA/DTL	Net DTA/DTL are value relevant to predict future stock returns	✓	✓	–	–	–
*Primary research area: Earnings management (Contribution to key issues C, D and E)*										
Cassell et al. (2015)	US GAAP	2460 firm-year obs	2008–2010	Total DTA, total VA	The extent of accruals-based earnings management is higher if firms do not provide transparent disclosure about DTA, and VA	–	–	✓	–	–
Kapoutsou et al. (2015)	IFRS	584 firm-year obs	2005–2008	Total DTE	Total DTE are positively related to the level of discretionary accruals	–	–	✓	–	–
Hanlon et al. (2012)	US GAAP	17,613 firm-year obs	2000–2007	Total DTE	Firms with higher DTE are associated with higher audit fees, as increased DTE signal earnings management	–	–	✓	–	–
Zhou (2016)	US GAAP	2470 firm-year obs	2007–2011	Total net DTE, large pos./neg. DTE	Net DTE are negatively related to earnings persistence	–	–	–	✓	–
Junqueira and Nakao (2013)	Brazil GAAP	51 firm-year obs	2000–2005	Total DTA, components DTA	Brazilian financial institutions use deferred taxes to attain the minimum equity capital level required by the Basel accord	–	–	–	✓	–
Kasipillai and Mahenthiran (2013)	Malaysian GAAP	317 firms	2005–2008	Total/change rate DTE	DTE are used to avoid earnings decline. The extent to which earnings are managed depends on the board structure of the respective firm	–	–	–	✓	–
Richardson and Leung (2011)	Hong Kong GAAP	542 firm-year obs	2005	Total DTE	DTE are positively related to earnings management to avoid earnings decline, loss reporting and to meet or beat analysts' forecast	–	–	–	✓	–
Christensen et al. (2008)	US GAAP	888 firms	1996–1998	Total/components DTA	DTA and components of DTA are not systematically used to increase earnings	–	–	–	✓	–
Skinner (2008)	Japanese GAAP	86 banks	1998–2003	Total DTA/DTL, VA	Japanese banks have used DTA to rearch the regulatory capital	–	–	–	✓	–
Frank and Rego (2006)	US GAAP	2243 firm-year obs	1993–2002	Total DTA, VA	The VA is used to smooth earnings towards analysts' forecast but not to reach other earnings targets	–	–	–	✓	–
Chao et al. (2004)	US GAAP	2145 firm-year obs	1996–2000	Total VA	The VA are not systematically used to increase earnings	–	–	–	✓	–
Dhaliwal et al. (2004)	US GAAP	4656 firm-year obs	1986–1999	Total DTE	Firms that manage earnings towards analysts' forecast have higher DTE	–	–	–	✓	–
Holland, Jackson (2004)	UK-GAAP	116 firm-year obs	1991–1992	Under-/overprovision net DTA/DTL	Net DTA/DTL are used to manage earnings (smooth profits)	–	–	–	✓	–
Phillips et al. (2004)	US GAAP	396 firm-year obs	1994–2000	Components DTL, change DTL	Changes in the net DTL component (related to revenue/expenses) can be used to detect earnings management	–	–	–	✓	–
Phillips et al. (2003)	US GAAP	2785 firm-year obs	1994–2000	Total DTE	DTE are useful to detect (upward) earnings management	–	–	–	✓	–
Schrand and Wong (2003)	US GAAP	190 firm-year obs	1993–1998	Total DTA, VA	Bank managers use the VA to smooth earnings and to manage earnings upwards	–	–	–	✓	–
Stammerjohan and Hall (2003)	US GAAP	949 firm-year obs	1977–1996	Total DTE	There is no evidence of earnings management due to DTE	–	–	–	✓	–
Dechow et al. (2011)	US GAAP	2190 misstatements	1982–2005	Total DTE	DTE are not able to predict misstatements	–	–	–	–	✓
Badertscher et al. (2009)	US GAAP	8099 firm-year obs	1997–2002	Total DTA, components DTA	DTA, especially DTA resulting from tax loss carryforwards are used to manage earnings upwards	–	–	–	–	✓
Wilson (2009)	US GAAP	215 firms	1972–2002	Total DTE	Investors interpret large DTE as signal for earnings management, and low earnings quality	–	–	–	–	✓
Ettredge et al. (2008)	US GAAP	220 firms	1992–2002	Total DTE	DTE are increased in the year before fraudulent accounting	–	–	–	–	✓
Cho et al. (2006)	New Zealand GAAP	81 audit cases	1991–2000	Total DTE	Proposed audit adjustments are positively related to DTE	–	–	–	–	✓
Mills (1998)	US GAAP	1545 firm-year obs	1982–1992	Total DTE	DTE are a good predictor for upward earnings management	–	–	–	–	✓
*Primary research area: value relevance and earnings management (Contribution to key issues A, B, C, D and E)*										
Herbohn et al. (2016)	Australian GAAP	2651 firm-year obs	1998–2008	Components DTA, VA	Dependent on the situation, the recognition of DTA and VA can convey information about lower earnings quality or future profitability	–	✓	–	✓	–
Jackson (2015)	US GAAP	44,658 firm-year obs	1973–2006	Total DTE	Inconsistent results concerning the relation between DTE and tax expense changes and earnings management	✓	–	✓	✓	–
Blaylock et al. (2012)	US GAAP	21,043 firm-year obs	1993–2005	Large/small DTE	Large positive or large negative DTE are associated to earnings management and tax avoidance	✓	–	✓	–	–
Herbohn et al. (2010)	Australian GAAP	1205 firm-year obs	1999–2005	Total DTA, components DTA	DTA resulting from tax loss carryforwards are used to manage earnings upwards towards analyst forecasts	✓	–	–	✓	–
Kumar and Visvanathan (2003)	US GAAP	256 firms	1994–1998	Total DTA/DTL, VA	The VA conveys private information about the future profitability. The VA is no instrument to manage earnings	–	✓	–	✓	–
Behn et al. (1998)	US GAAP	322 firms	1993	Total DTA, VA	The VA is set in accordance with the economic circumstances of the firms, and legal requirements of SFAS 107	✓	–	–	✓	–

*DTA* deferred tax assets, *DTL* deferred tax liabilities, *DTE* deferred tax expenses, *VA* valuation allowance

A—Value relevance (Forecast relevance), B—Value relevance (Valuation relevance), C—Earnings management (Mis-/re-statements), D—Earnings management (Thresholds), E—Earnings management (Accruals)

4.

## Footnotes

1 It should be noted that earnings management comprises accounting choices and “real” earnings management. The latter relates to the timing of real decisions and the structuring of transactions (Healy and Wahlen 1999; Zamri et al. 2013). While “real” earnings management can affect deferred taxes, it is hard to detect in general and almost impossible to detect when focusing on deferred taxes. None of the empirical studies in our review focuses on “real” earnings management.

2 Due to the high number of influential articles published in the first decade, possible one-time effects due to the introduction of the standard SFAS No. 109 (ASC 740) in 1992 could be overweighed, especially as four of the most cited articles—Amir et al. (1997), Amir and Sougiannis (1999), Miller and Skinner (1998), and Ayers (1998)—have used similar data from the introduction years of SFAS No. 109 (ASC 740). The narrative synthesis of the articles strengthens the result presented here that early published US studies are more often considered, even though more recent results exist. Regardless, a citation analysis is generally biased toward older publications, as they have had more occasions to be cited than newer ones (Zupic and Čater 2015).

## Declarations

### Availability of data and material

All data available from Anna Görlitz.

## References

[CR1] Amir E, Sougiannis T (1999). Analysts’ interpretation and investors’ valuation of tax carryforwards. Contemp Account Res.

[CR2] Amir E, Kirschenheiter M, Willard K (1997). The valuation of deferred taxes. Contempl Accout Res.

[CR3] Arcelus FJ, Mitra D, Srinivasan G (2005). On the incidence of deferred taxes, intangibles and non-linearities in the relationship between Tobin’s Q and ROI. J Econ Bus.

[CR4] Atwood T, Drake MS, Myers LA (2010). Book-tax conformity, earnings persistence and the association between earnings and future cash flows. J Account Econ.

[CR5] Ayers BC (1998). Deferred tax accounting under SFAS No. 109: an empirical investigation of its incremental value-relevance relative to APB No. 11. Account Rev.

[CR6] Badenhorst WM, Ferreira PH (2016). The financial crisis and the value-relevance of recognised deferred tax assets. Aust Account Rev.

[CR7] Badertscher BA, Phillips JD, Pincus M, Rego SO (2009). Earnings management strategies and the trade-off between tax benefits and detection risk: to conform or not to conform?. Account Rev.

[CR8] Barth ME, Cram DP, Nelson KK (2001). Accruals and the prediction of future cash flows. Account Rev.

[CR9] Barth ME, Beaver WH, Landsman WR (2001). The relevance of the value relevance literature for financial accounting standard setting: another view. J Account Econ.

[CR10] Bauman MP, Das S (2004). Stock market valuation of deferred tax assets: evidence from internet firms. J Bus Financ Account.

[CR11] Bauman MP, Shaw KW (2008). The usefulness of disclosures of untaxed foreign earnings in firm valuation. J Am Tax Assoc.

[CR12] Bauman MP, Shaw KW (2016). Balance sheet classification and the valuation of deferred taxes. Res Account Regul.

[CR13] Beaver WH (2002). Perspectives on recent capital market research. Account Rev.

[CR14] Beaver WH, Dukes RE (1972). Interperiod tax allocation, earnings expectations, and the behavior of security prices. Account Rev.

[CR15] Beechy TH (2007). The make-believe world of future income taxes. Account Perspect.

[CR16] Behn BK, Eaton TV, Williams JR (1998). The determinants of the deferred tax allowance account under SFAS No. 109. Account Horiz.

[CR17] Beisland LA (2009). A review of the value relevance literature. Open Bus J.

[CR18] Biddle GC, Chen P, Zhang G (2001). When capital follows profitability: non-linear residual income dynamics. Rev Account Stud.

[CR19] Blaylock B, Shevlin T, Wilson RJ (2012). Tax avoidance, large positive temporary book-tax differences, and earnings persistence. Account Rev.

[CR20] Block JH, Fisch C (2020). Eight tips and questions for your bibliographic study in business and management research. Manag Rev Q.

[CR21] Breitkreuz R (2012). Latente steuern und earnings management. Z Betriebswirtschaft.

[CR22] Brouwer A, Naarding E (2018). Making deferred taxes relevant. Account Eur.

[CR23] Cassell CA, Myers LA, Seidel TA (2015). Disclosure transparency about activity in valuation allowance and reserve accounts and accruals-based earnings management. Account Organ Soc.

[CR24] Chaney PK, Jeter DC (1994). The effect of deferred taxes on security prices. J Account Audit Finance.

[CR25] Chang C, Herbohn K, Tutticci I (2009). Market’s perception of deferred tax accruals. Account Finance.

[CR26] Chao CL, Kelsey RL, Horng SM, Chiu CY (2004). Evidence of earnings management from the measurement of the deferred tax allowance account. Eng Econ.

[CR27] Chattopadhyay S, Arcelus FJ, Srinivasan G (1997). Deferred taxes and bond ratings: a Canadian case. J Bus Finance Account.

[CR28] Chen KC, Schoderbek MP (2000). The 1993 tax rate increase and deferred tax adjustments: a test of functional fixation. J Account Res.

[CR29] Chen KC, Danielson MG, Schoderbek MP (2003). Analysts’ interpretation of transitory earnings components: evidence from forecast revisions after disclosure of the 1993 deferred tax adjustment. J Account Audit Finance.

[CR30] Cheung JK, Krishnan GV, Chung-ki M (1997). Does interperiod income tax allocation enhance prediction of cash flows?. Account Horiz.

[CR31] Chi SS, Pincus M, Teoh SH (2014). Mispricing of book-tax differences and the trading behavior of short sellers and insiders. Account Rev.

[CR32] Chludek AK (2011). Perceived versus actual cash flow implications of deferred taxes—an analysis of value relevance and reversal under IFRS. J Int Account Res.

[CR33] Cho J, Wong J, Wong N (2006). Book-tax differences and Inland Revenue audit adjustments in New Zealand. J Bus Finance Account.

[CR34] Christensen TE, Paik GH, Stice EK (2008). Creating a bigger bath using the deferred tax valuation allowance. J Bus Finance Account.

[CR35] Citron DB (2001). The valuation of deferred taxation: Evidence from the UK partial provision approach. J Bus Finance Account.

[CR36] Clark WR, Clark LA, Raffo DM (2020). Extending fisch and block’s (2018) tips for a systematic review in management and business literature. Manag Rev Q.

[CR37] Coase RH (1990). Accounting and the theory of the firm. J Account Econ.

[CR38] Colley R, Rue J, Volkan A (2009). Continuing case against inter-period tax allocation. J Bus Econ Res.

[CR39] Comprix J, Graham RC, Moore JA (2011). Empirical evidence on the impact of book-tax differences on divergence of opinion among investors. J Am Tax Assoc.

[CR40] Crabtree A, Maher JJ (2009). The influence of differences in taxable income and book income on the bond credit market. J Am Tax Assoc.

[CR41] Dechow PM, Skinner DJ (2000). Earnings management: reconciling the views of accounting academics, practitioners, and regulators. Account Horiz.

[CR42] Dechow PM, Ge W, Larson CR, Sloan RG (2011). Predicting material accounting misstatements. Contemp Account Res.

[CR43] Deloitte (2020) 1 Japan Tax and Legal Inbound Newsletter. https://www2.deloitte.com/content/dam/Deloitte/jp/Documents/tax/bt/jp-bt-japan-tax-legal-inbound-July2020-no58.pdf. Accessed 28 July 2021.

[CR44] Denyer D, Tranfield D, Buchanan D, Bryman A (2009). Producing a systematic review. The Sage handbook of organizational research methods.

[CR45] Dhaliwal DS, Gleason CA, Mills LF (2004). Last-chance earnings management: using the tax expense to meet analysts' forecasts. Contemp Account Res.

[CR46] Dhaliwal DS, Kaplan SE, Laux RC, Weisbrod E (2013). The information content of tax expense for firms reporting losses. J Account Res.

[CR47] Dichev ID (2008). On the balance sheet-based model of financial reporting. Accout Horiz.

[CR48] Dichev ID, Graham JR, Harvey CR, Rajgopal S (2013). Earnings quality: evidence from the field. J Account Econ.

[CR49] DPR (2015) 10th Anniversary of FREP. https://www.frep.info/docs/dpr_10_jahre/dpr_jubilaeumsbroschuere.pdf. Accessed 28 July 2021.

[CR50] Edwards A (2018). The deferred tax asset valuation allowance and firm creditworthiness. J Am Tax Assoc.

[CR51] EFRAG (2013) Improving the Financial Reporting of Income Tax, Feedback Statement. http://old.efrag.org/files/ProjectDocuments/Proactive%20-%20Income%20Taxes/130208_Income_Tax_Feedback_Statement.pdf. Accessed 28 July 2021.

[CR52] EFRAG (2019) 14/11/2019-EFRAG final comment letter on the IASB exposure draft ED/2019/5 Deferred Tax Related to Assets and Liabilities Arising from a Single Transaction-Proposed amendments to IAS 12. https://www.efrag.org/News/Project-394/EFRAG-final-comment-letter-on-the-IASB-exposure-draft-ED20195-Deferred-Tax-Related-to-Assets-and-Liabilities-Arising-from-a-Single-Transaction---Proposed-amendments-to-IAS-12. Accessed 28 July 2021.

[CR53] Eiler L, Kutcher L (2016). SEC comment letters related to permanently reinvested earnings. Adv Account.

[CR54] Ettredge ML, Sun L, Lee P, Anandarajan AA (2008). Is earnings fraud associated with high deferred tax and/or book minus tax levels?. Audit J Pract Theory.

[CR55] Ewert R, Wagenhofer A (2013) Accounting standards, earnings management, and earnings quality. Available at SSRN. http://dx.doi.org/10.2139/ssrn.2068134

[CR56] EY (2018) SEC Comments and trends—an analysis of current reporting issues. https://www.ey.com/publication/vwluassetsdld/seccommentstrends_06976-191us_18september2019/$file/seccommentstrends_06976-191us_18september2019.pdf?OpenElement. Accessed 28 July 2021.

[CR57] Fama EF (1980). Agency problems and the theory of the firm. J Polit Econ.

[CR58] FASB (2015) Income Taxes (Topic 740)—Balance sheet classification of deferred taxes. https://www.fasb.org/jsp/FASB/Document_C/DocumentPage?cid=1176167636650&acceptedDisclaimer=true. Accessed 28 July 2021.

[CR59] FASB (2019) Accounting Standards Update 2019–12—income taxes (Topic 740): simplifying the accounting for income taxes. https://www.fasb.org/cs/ContentServer?c=FASBContent_C&cid=1176174066395&d=&pagename=FASB%2FFASBContent_C%2FCompletedProjectPage. Accessed 28 July 2021.

[CR60] Feltham GA, Ohlson JA (1995). Valuation and clean surplus accounting for operating and financial activities. Contemp Account Res.

[CR61] Feltham GA, Pae J (2000). Analysis of the impact of accounting accruals on earnings uncertainty and response coefficients. J Account Audit Finance.

[CR62] Fisch C, Block J (2018). Six tips for your (systematic) literature review in business and management research. Manang Rev Q.

[CR63] Frank MM, Rego SO (2006). Do managers use the valuation allowance account to manage earnings around certain earnings targets?. J Am Tax Assoc.

[CR64] Gaeremynck A, Van De Gucht L (2004). The recognition and timing of deferred tax liabilities. J Bus Finance Account.

[CR65] Givoly D, Hayn C (1992). The valuation of the deferred tax liability: evidence from the stock market. Account Rev.

[CR66] Gordon EA, Joos PR (2004). Unrecognized deferred taxes: evidence from the UK. Account Rev.

[CR67] Graham RC, Moore JA (2018). The mitigation of high-growth-related accounting distortions after Sarbanes-Oxley. Res Account Regul.

[CR68] Graham JR, Ready JS, Shackelford DA (2012). Research in accounting for income taxes. J Account Econ.

[CR69] Guay WR, Kothari SP, Watts RL (1996). A market-based evaluation of discretionary accrual models. J Account Res.

[CR70] Hanlon M (2005). The persistence and pricing of earnings, accruals, and cash flows when firms have large book-tax differences. Account Rev.

[CR71] Hanlon M, Heitzman S (2010). A review of tax research. J Account Econ.

[CR72] Hanlon M, Krishnan GV, Mills LF (2012). Audit fees and book-tax differences. J Am Tax Assoc.

[CR73] Hanlon D, Navissi F, Soepriyanto G (2014). The value relevance of deferred tax attributed to asset revaluations. J Contemp Account Econ.

[CR74] Hanna JD, Li Z, Shaw W (2019). Banks’ deferred tax assets during the financial crisis. Rev Quant Finance Account.

[CR75] Hart C (2001). Doing a literature search: a comprehensive guide for the social sciences.

[CR76] Harumova A (2017). The economic function of deferred taxes.

[CR77] Haupt M, Ismer R (2013). The EU emissions trading system under IFRS–towards a ‘true and fair view’. Account Eur.

[CR78] Healy PM, Wahlen JM (1999). A review of the earnings management literature and its implications for standard setting. Account Horiz.

[CR79] Herbohn K, Tutticci I, Khor PS (2010). Changes in unrecognised deferred tax accruals from carry-forward losses: earnings management or signalling?. J Bus Financ Account.

[CR80] Herbohn K, Tutticci I, Tan Z (2016). The market response to beating after-tax earnings targets revisited using analysts’ pre-tax earnings forecasts and concurrent tax note disclosures. J Bus Financ Account.

[CR81] Holland K, Jackson RH (2004). Earnings management and deferred tax. Account Bus Res.

[CR82] Holthausen RW, Watts RL (2001). The relevance of the value-relevance literature for financial accounting standard setting. J Account Econ.

[CR83] Huang DF, Chang ML (2016). Do auditor-provided tax services improve the relation between tax-related internal control and book-tax differences?. Asia-Pac J Account Econ.

[CR84] Huang DF, Wang CL (2013). Book-tax differences and earnings quality for the banking industry: evidence from Taiwan. Pac Account Rev.

[CR85] IASB (2016a) IAS 12 income taxes research project. http://archive.ifrs.org/Meetings/MeetingDocs/IASB/2016/May/AP19A-Income-Taxes.pdf. Accessed 28 July 2021

[CR86] IASB (2016b) IAS 12 income taxes research project. http://archive.ifrs.org/Meetings/MeetingDocs/IASB/2016/May/AP19C-Income-Taxes.pdf. Accessed 28 July 2021

[CR87] IASB (2016c) IASB issues narrow-scope amendments to IAS 12 Income Taxes. https://www.ifrs.org/news-and-events/2016/01/iasb-issues-narrow-scope-amendments-to-ias-12-income-taxes/. Accessed 28 July 2021

[CR88] IASB (2017) Better communication in financial reporting—making disclosures more meaningful. https://www.ifrs.org/-/media/project/disclosure-initative/better-communication-making-disclosures-more-meaningful.pdf. Accessed 28 July 2021

[CR89] IASB (2018) IAS 12 Income taxes deferred tax–tax base of assets and liabilities. www.ifrs.org/-/media/feature/meetings/2018/october/iasb/ap12c-ias12.pdf. Accessed 28 July 2021

[CR90] IASB (2019) Deferred Tax related to Assets and Liabilities arising from a Single Transaction Proposed amendments to IAS 12. https://cdn.ifrs.org/-/media/project/deferred-tax-related-to-assets-and-liabilities-arising-from-a-single-transaction/ed-deferred-tax-related-to-assets-and-liabilities-ias-12.pdf. Accessed 28 July 2021

[CR91] IASB (2020) IAS 12—deferred tax related to assets and liabilities arising from a single transaction. https://www.iasplus.com/en/meeting-notes/ifrs-ic/2020/september/ias-12. Accessed 28 July 2021

[CR92] Inamura Y, Okuda S (2017). Deferred taxes and cost of debt: evidence from Japan. Asia-Pac J Account Econ.

[CR93] IRD (2021) COVID-19 Depreciation and low-value assets. https://www.ird.govt.nz/covid-19/business-and-organisations/specific-income-tax-issues/depreciation-and-low-value-assets. Accessed 28 July 2021

[CR94] Jackson M (2015). Book-tax differences and future earnings changes. J Am Tax Assoc.

[CR95] Johnston D, Kutcher L (2016). Do stock-based compensation deferred tax assets provide incremental information about future tax payments?. J Am Tax Assoc.

[CR96] Jones JJ (1991). Earnings management during import relief investigations. J Account Res.

[CR97] Junqueira MADR, Nakao SH (2013). The role of deferred tax in the regulatory capital of Brazilian financial institutions. Revista Contabilidade Financas.

[CR98] Kapoutsou E, Tzovas C, Chalevas C (2015). Earnings management and income tax evidence from Greece. Corp Ownersh Control.

[CR99] Kasipillai J, Mahenthiran S (2013). Deferred taxes, earnings management, and corporate governance: Malaysian evidence. J Contemp Account Econ.

[CR100] Kovermann J, Velte P (2019). Net deferred tax assets and the long-run performance of initial public offerings. Corp Ownersh Control.

[CR101] Kovermann J, Velte P (2021). CSR and tax avoidance: a review of empirical research. Corp Ownersh Control.

[CR102] KPMG (2020) China: Tax developments in response to COVID-19. https://home.kpmg/xx/en/home/insights/2020/04/china-tax-developments-in-response-to-covid-19.html. Accessed 28 July 2021

[CR103] KPMG (2021) Coronavirus (COVID-19) tax developments. https://home.kpmg/xx/en/home/insights/2020/04/taxnewsflash-coronavirus-covid-19-developments.html. Accessed 28 July 2021

[CR104] Kraft A, Lopatta K (2016). Auditor fees, discretionary book-tax differences, and tax avoidance. Int J Econ Account.

[CR105] Krishnan GV, Visvanathan G (2011). Is there an association between earnings management and auditor-provided tax services?. J Am Tax Assoc.

[CR106] Kumar KR, Visvanathan G (2003). The information content of the deferred tax valuation allowance. Account Rev.

[CR107] Lambert RA (2006). Agency theory and management accounting. Handb Manag Account Res.

[CR108] Laurion H, Lawrence A, Ryans JP (2017). US audit partner rotations. Account Rev.

[CR109] Laux RC (2013). The association between deferred tax assets and liabilities and future tax payments. Account Rev.

[CR110] Legoria J, Sellers KF (2005). The analysis of SFAS No. 109’s usefulness in predicting future cash flows from a conceptual framework perspective. Res Account Regul.

[CR111] Lennox C, Lisowsky P, Pittman J (2013). Tax aggressiveness and accounting fraud. J Account Res.

[CR112] Lev B, Nissim D (2004). Taxable income, future earnings, and equity values. Account Rev.

[CR113] Lynn SG, Seethamraju C, Seetharaman A (2008). Incremental value relevance of unrecognized deferred taxes: evidence from the United Kingdom. J Am Tax Assoc.

[CR114] Marques AVC, Costa PDS, Silva PR (2016). The relevance of the informational content of book-tax differences for predicting future income: Evidence from Latin American countries. Revista Contabilidade Finanças.

[CR115] McGuire ST, Neuman SS, Olson AJ, Omer TC (2016). Do investors use prior tax avoidance when pricing tax loss carryforwards?. J Am Tax Assoc.

[CR116] Mear K, Bradbury M, Hooks J (2019). The ability of deferred tax to predict future tax. Account Finance.

[CR117] Miller GS, Skinner DJ (1998). Determinants of the valuation allowance for deferred tax assets under SFAS No. 109. Account Rev.

[CR118] Mills LF (1998). Book-tax differences and internal revenue service adjustments. J Account Res.

[CR119] Moore JA, Xu L (2018). Book-tax differences and costs of private debt. Adv Account.

[CR120] Morton EF (2019). A historical review of the rise of tax effect accounting as a financial reporting norm. Account Hist.

[CR121] Murdoch B, Krause P, Guy P (2015). An analysis of using time-series current and deferred income tax expense to forecast income taxes paid. J Appl Bus Res.

[CR122] Neifar S, Utz S (2019). The effect of earnings management and tax aggressiveness on shareholder wealth and stock price crash risk of German companies. J Appl Account Res.

[CR123] Ohlson JA (1995). Earnings, book values, and dividends in equity valuation. Contemp Account Res.

[CR124] Phillips J, Pincus M, Rego SO (2003). Earnings management: new evidence based on deferred tax expense. Account Rev.

[CR125] Phillips JD, Pincus M, Rego SO, Wan H (2004). Decomposing changes in deferred tax assets and liabilities to isolate earnings management activities. J Am Tax Assoc.

[CR126] Poterba JM, Rao NS, Seidman JK (2011). Deferred tax positions and incentives for corporate behavior around corporate tax changes. Natl Tax J.

[CR127] PWC (2016) What do investors want to see in company tax disclosures? https://www.pwc.com/gx/en/audit-services/corporate-reporting/investor-views/pdf/investor-view-tax.pdf. Accessed 28 July 2021

[CR128] Rego SO, Wilson R (2012). Equity risk incentives and corporate tax aggressiveness. J Account Res.

[CR129] Richardson G, Leung S (2011). Family ownership control and earnings management: evidence from Hong Kong firms. Corp Ownersh Control.

[CR130] Samara AD (2014). Assessing the relevance of deferred tax items: Evidence from loss firms during the financial crisis. J Econ Asymmetries.

[CR131] Sankar MR, Subramanyam KR (2001). Reporting discretion and private information communication through earnings. J Account Res.

[CR132] Schrand CM, Wong MF (2003). Earnings management using the valuation allowance for deferred tax assets under SFAS No. 109. Contemp Account Res.

[CR133] Schultz SM, Johnson RT (1998). Income tax allocation: the continuing controversy in historical perspective. Account Hist J.

[CR134] SCImago (2019) SCImago Journal and Country Rank. https://www.SCImagojr.com/journalrank.phpte. Accessed 28 July 2021

[CR135] Skinner DJ (2008). The rise of deferred tax assets in Japan: the role of deferred tax accounting in the Japanese banking crisis. J Account Econ.

[CR136] Stammerjohan WW, Hall SC (2003). Legal costs and accounting choices: another test of the litigation hypothesis. J Bus Fin Account.

[CR137] Tranfield D, Denyer D, Smart P (2003). Towards a methodology for developing evidence-informed management knowledge by means of systematic review. Br J Manag.

[CR138] Wahab NSA, Holland K (2015). The persistence of book-tax differences. Br Account Rev.

[CR139] Wilson RJ (2009). An examination of corporate tax shelter participants. Account Rev.

[CR140] Wong J, Wong N, Naiker V (2011). Comprehensive versus partial deferred tax liabilities and equity market values. Account Finance.

[CR141] Zamri N, Rahman RA, Isa NSM (2013). The impact of leverage on real earnings management. Procedia Econ Finance.

[CR142] Zeng T (2003). The valuation of loss carryforwards. Can J Adm Sci.

[CR143] Zhou M (2016). Does accounting for uncertain tax benefits provide information about the relation between book-tax differences and earnings persistence?. Rev Account Finance.

[CR144] Zupic I, Čater T (2015). Bibliometric methods in management and organization. Organ Res Methods.

